# Integrated multi-omics analysis unveils microbiota-metabolite-host interactions and novel biomarkers for early diabetic kidney disease diagnosis

**DOI:** 10.3389/fimmu.2026.1781013

**Published:** 2026-03-09

**Authors:** Tao Jiang, Jialin Deng, Xiaojuan Hu, Dongsheng Yao, Qingguang Chen, Ruomeng Hu, Xuxiang Ma, Liping Tu, Xin Tan, Wang Yuan, Lizhuang Ma, Ji Cui, Hao Lu, Jiatuo Xu

**Affiliations:** 1 School of Traditional Chinese Medicine, Shanghai University of Traditional Chinese Medicine, Shanghai, China; 2 Department of Endocrinology, Shuguang Hospital Affiliated to Shanghai University of Traditional Chinese Medicine, Shanghai, China; 3 School of Computer Science and Technology, East China Normal University, Shanghai, China; 4 School of Computer Science, Shanghai Jiao Tong University, Shanghai, China

**Keywords:** biomarkers, diabetic kidney disease, early diagnosis, East Asian, machine learning, Mendelian randomization, metabolites

## Abstract

**Background:**

Diabetic kidney disease (DKD) is a leading cause of end-stage renal disease (ESRD), and its early diagnosis remains a major global challenge because conventional biomarkers lack sensitivity. The East Asian population is characterized by distinct genetic, environmental, and lifestyle factors that may influence the development and progression of DKD, highlighting the importance of population-specific research. The primary objective of this study was to apply a multi-omics strategy, including Mendelian randomization (MR) analysis, within an East Asian cohort to investigate potential causal relationships among microbiota, metabolites, and DKD, with the aim of identifying candidate biomarkers relevant to this population. Secondary objectives included the analysis of clinical samples from East Asian participants to characterize microbiota composition, metabolomic profiles, and tongue image features (TIFs), as well as the development of machine learning (ML) models to distinguish patients with type 2 diabetes mellitus (T2DM) from those with DKD.

**Methods:**

MR analysis was performed to investigate potential causal associations between more than 190 microbiota taxa and 404 differential metabolites in relation to DKD within the East Asian cohort. Clinical samples (n = 535) were collected from East Asian individuals and analyzed for microbiota composition, metabolomic profiling, and TIFs. Subsequently, ML models were constructed to differentiate patients with T2DM from those with DKD in this cohort.

**Results:**

MR analysis identified significant associations between specific microbiota taxa (e.g., Haemophilus-A, TM7x, Lachnoanaerobaculum, and Bacteroides) and metabolites (e.g., tyrosine and glutamine) in relation to DKD within the East Asian cohort. However, the causal nature of these associations requires further experimental or longitudinal validation. Clinical analyses revealed microbial dysbiosis in patients with DKD, including a 2.5-fold increase in Klebsiella and a 60% reduction in Faecalibaculum and Dubosiella. Metabolomic profiling demonstrated alterations in branched-chain amino acids (BCAAs) and fatty acids. Integrated multi-omics analysis suggested complex interactions among microbiota and metabolites that may contribute to DKD progression. The ML models achieved an accuracy exceeding 90% in distinguishing T2DM from DKD in the East Asian cohort.

**Conclusion:**

Multi-omics integration combined with ML may provide candidate biomarkers for the early detection of DKD in the East Asian population. These approaches could improve the accuracy of non-invasive diagnosis and support the development of personalized management strategies. Nevertheless, further studies are required to validate the identified associations and confirm their clinical applicability in real-world East Asian settings.

## 1 Introduction

Diabetic kidney disease (DKD) is a form of chronic kidney disease (CKD) and a common complication of diabetic microangiopathy, characterized by structural and functional renal impairment (1). Its clinical manifestations, including significant proteinuria, hypertension, and edema, typically appear during the intermediate to advanced stages of type 2 diabetes mellitus (T2DM). DKD is the leading cause of end-stage renal disease (ESRD) worldwide (2). Current diagnostic criteria for DKD are primarily based on a decline in glomerular filtration rate (GFR) or an increase in the urinary albumin-to-creatinine ratio (UACR). However, these indicators have limited sensitivity and specificity in the early stages of renal injury (3). This limitation underscores the need to identify novel biomarkers derived from the underlying pathogenic mechanisms of DKD to improve early diagnosis, disease monitoring, therapeutic decision-making, and prognostic evaluation.

Microbiota play critical roles in regulating immunity, metabolism, and disease progression. Microbial dysbiosis is increasingly recognized as a risk factor for various chronic diseases, including T2DM, gastrointestinal (GI) cancers, and neurological disorders (4–6). The human digestive tract functions as a continuous ecological system, with the oral cavity serving as the primary entry point for nutrients and microorganisms. Compared with the gut microbiota, the oral microbiota may act as a more sensitive early indicator of metabolic alterations, such as hyperglycemia, because of its direct exposure to systemic circulation and rapid response to physiological changes (7, 8). Importantly, the oral microbiota does not function independently. It interacts dynamically with the gut microbiota through the oral–gut axis, whereby dysbiotic oral bacteria may translocate to the intestine and contribute to systemic inflammation and metabolic endotoxemia (9). Previous studies on DKD have largely focused on the gut microbiome. However, most investigations have emphasized overall compositional alterations and their correlations with DKD progression, while paying limited attention to complex host–microbiota–metabolite interactions or to the potential diagnostic value of the oral microbiome, particularly when integrated with gut microbiome data. In DKD, renal dysfunction may disrupt microbial homeostasis, and the resulting dysbiosis can further aggravate kidney injury through the production of uremic toxins and other harmful metabolites, potentially contributing to irreversible damage. In addition, the tongue—an extension of the oral mucosa—provides a visible interface between the host and the oral microbial ecosystem. Tongue coating characteristics and body color (e.g., yellow coating, red body) may reflect underlying microbial dysbiosis and inflammatory status rather than serving solely as subjective clinical observations (10). Oral microbiota profiling combined with tongue imaging may therefore offer complementary diagnostic information. The oral cavity represents the initial site of exposure to metabolic and immunological stimuli, making oral microbial alterations potentially sensitive indicators of early-stage disease. Tongue imaging provides a non-invasive approach to visualize phenotypic features associated with oral microbial status. Integrating these modalities may yield a more comprehensive understanding of the oral microbial ecosystem and its relationship with DKD. Strategies aimed at restoring microbial balance—including dietary modification, fecal microbiota transplantation (FMT), administration of probiotics or prebiotics, and reduction of uremic toxin accumulation—may help delay DKD progression (11–13).

The integration of microbiome research and metabolomics has become a major focus in endocrine disease studies. Metabolomic analyses have identified stepwise alterations in bile acid metabolism among patients with DKD (14). Differential metabolites identified through metabolomics may serve as potential biomarkers for early diagnosis, therapeutic target discovery, mechanistic investigations, and prognostic assessment (15, 16). Although multi-omics approaches are widely applied in endocrine and metabolic disease research, clinical studies investigating oral–gut characteristic biomarkers in DKD remain limited. Moreover, the biological mechanisms underlying the progression from T2DM to DKD have not been fully elucidated.

Machine learning (ML), an advanced artificial intelligence (AI) technique, has gained increasing attention in microbiome research. Compared with conventional logistic regression, ML algorithms designed for multimodal data integration—such as Random Forest, Extreme Gradient Boosting (XGBoost), and Naïve Bayes—provide improved performance in handling high-dimensional and complex datasets (17). ML approaches are increasingly used in disease diagnosis and early risk prediction for metabolic disorders (18–20). Owing to robust feature selection and extraction capabilities, ML is particularly well suited for identifying microbial biomarkers and modeling complex biological interactions (21, 22).

In contrast to previous studies that have focused solely on the gut microbiome or metabolomics, this study introduces a pioneering multi-modal approach by incorporating both the oral microbiome and tongue imaging. It is the first to comprehensively explore the causal relationships among oral and gut microbiota, blood metabolites, and DKD, utilizing Mendelian randomization (MR) as a gateway. This approach provides novel insights into the intricate interplay among these factors, combining oral and gut microbiota analysis, blood metabolomics, and tongue imaging into a unified multi-omics framework. MR analysis was used to investigate the links between oral and gut microbiota, blood metabolites, and DKD, while systematically examining the relationship between these microbiota and paired blood metabolites in 535 subjects. Additionally, machine learning methods were employed to develop a diagnostic model for DKD. Full-length 16S rRNA gene sequencing enabled detailed analysis of microbiota composition and diversity across various body sites, helping to identify DKD biomarkers within the microbiome and uncovering the biological mechanisms underlying the progression from T2DM to DKD.

## 2 Methods

### 2.1 Reanalysis of publicly available metabolomic data

#### 2.1.1 Mendelian randomization study design

**Figure 1:**
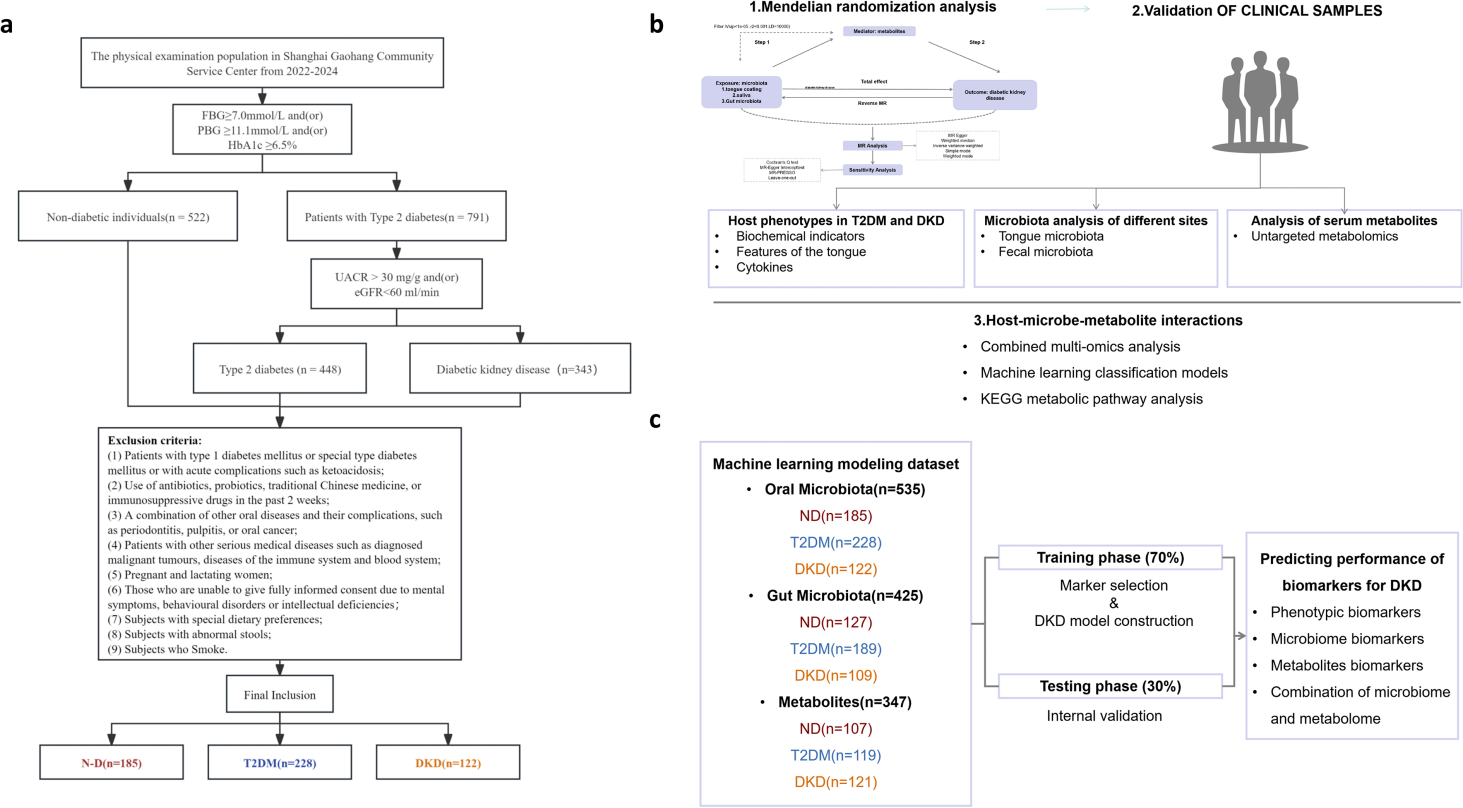
Flow Chart: **(a)** The clinical study included samples from individuals without diabetes, T2DM patients, and DKD patients. The number of elements in each dataset following quality filtering is depicted in the figure. **(b)** Multi-omics analysis workflow: (1) MR analysis of microorganisms and metabolites associated with different stages of DKD was initially performed using online datasets (upper left); (2) Clinical data were subsequently collected, and microbial characteristics were assessed via 16S sequencing. The correlation between microbial features and metabolites was analyzed. To better understand host-microbe interactions, associations between gut metabolites/microorganisms and host plasma metabolites, cytokines, and PBMC genes were explored. The mediating role of plasma cytokines in the relationship between microorganisms and metabolic markers was also assessed. The metabolic pathways linking T2DM to DKD were examined using the KEGG signaling pathway. Finally, a diagnostic classification model for DKD was developed using machine learning methods. **(c)** Workflow of the ML algorithm, including feature selection and model construction.

The overall study design is illustrated in **Figure 1b**. Published genome-wide association study (GWAS) summary statistics were first retrieved, including microbiota data from saliva, tongue, and gut; plasma metabolite profiles; and DKD-related phenotypes (**Supplementary Table 1**). Two-sample MR analysis was then conducted to evaluate the potential causal effects of microbiota and plasma metabolites from saliva, tongue, and gut on DKD. The objective was to identify microbial taxa and metabolites strongly associated with DKD risk. Finally, mediation analysis was performed to assess whether selected microbiota influenced DKD through the identified metabolites.

#### 2.1.2 Data sources

Oral and gut microbiota data were obtained from the China National GeneBank database (https://db.cngb.org/). Dataset CNP0001664 contains saliva and tongue microbiota data from East Asian populations, whereas CNP0000794 includes gut microbiota and blood metabolite data from the same population. Data release was authorized by the Ministry of Science and Technology of China (Project No. 2020BAT1137) (23). GWAS summary statistics covering 191 intestinal microbiota taxa (9 phyla, 16 classes, 19 orders, 30 families, 102 genera, and 15 species) were included for MR analysis. DKD GWAS data (Ebi-a-GCST90018612) were retrieved from the GWAS Catalog (https://gwas.mrcieu.ac.uk/), comprising 132,984 East Asian participants and 12,447,074 single nucleotide polymorphisms (SNPs).

#### 2.1.3 Instrumental variable selection

Three core assumptions for instrumental variables (IVs) were required for valid causal inference (1): IVs must be strongly associated with the exposure (2); IVs must not be associated with confounding factors (3); IVs must influence the outcome exclusively through the exposure (24).

Because genetic variants are randomly allocated during meiosis, analogous to randomization in controlled trials, MR reduces confounding and enhances causal inference robustness. SNPs were therefore selected as IVs for both exposures and outcomes using predefined criteria. Based on previous studies, SNPs associated with gut microbiota and plasma metabolites were selected at a significance threshold of *P* < 1 × 10^–5^. When hypersensitivity phenotype (HSP) was treated as the exposure, a more stringent threshold (*P* < 5 × 10^–6^) still yielded a sufficient number of IVs. To ensure adequate instrument strength and analytical feasibility, the threshold was adjusted accordingly while maintaining statistical rigor. To ensure independence among IVs and minimize linkage disequilibrium effects, clumping was performed using the TwoSampleMR R package with parameters set at R (2) < 0.001 and a clumping window of 10,000 kb (25). Instrument strength was evaluated using the F-statistic, and SNPs with F > 10 were retained to avoid weak instrument bias (26). SNPs meeting all selection criteria were included in the final MR analysis.

#### 2.1.4 Statistical analysis

Two-sample MR analyses were conducted using R software (version 4.3.2) with the packages TwoSampleMR, VariantAnnotation, and ieugwasr. Five MR methods were applied: inverse variance weighted (IVW), MR-Egger, weighted median, simple mode, and weighted mode. The IVW method was designated as the primary analytical approach because of its higher precision under valid instrument assumptions. Statistical significance was defined as *P* < 0.05. Odds ratios (ORs) were calculated to quantify effect sizes, with OR > 1 indicating a positive association and OR < 1 indicating a negative association. Heterogeneity was assessed using Cochran’s Q statistic. When significant heterogeneity was detected, MR-Egger regression was performed to evaluate horizontal pleiotropy. Leave-one-out sensitivity analysis was conducted to determine the influence of individual SNPs on causal estimates. Funnel and scatter plots were generated to visually assess potential pleiotropic effects. To control for multiple testing and reduce false-positive findings, false discovery rate (FDR) correction was applied. We also conducted bidirectional MR analyses to explore potential reverse causal relationships. Only results meeting the FDR-adjusted significance threshold were retained. This analytical framework minimized bias and strengthened the reliability of the causal estimates.

### 2.2 Clinical research participants

Participants were recruited between 2022 and 2024 from the Physical Examination Center and the Department of Endocrinology at Shuguang Hospital, affiliated with Shanghai University of Traditional Chinese Medicine. According to predefined inclusion and exclusion criteria, a total of 535 individuals were enrolled, including 185 non-diabetic (ND) controls, 228 patients with T2DM, and 122 patients with DKD (**Figure 1a**; **Table 1**). Written informed consent was obtained from all participants. The study protocol was approved by the Ethics Committee of Shuguang Hospital (Ethics No. ChiCTR2100043546).

**Table 1:** Inclusion and exclusion criteria for subjects.

Inclusion criteria
Age 20–80 years T2DM Diagnostic Criteria: Inclusion required meeting one or more of the following: 1) Random blood glucose ≥ 11.1 mmol/L; 2) FBG ≥ 7.0 mmol/L; 3) PBG ≥ 11.1 mmol/L; 4) HbA1C ≥6.5%. DKD Diagnostic Criteria: UACR > 30 mg/g and/or eGFR < 60 mL·min^-1^·(1.73 m^2^) ^-1^ lasting more than 3 months
Exclusion criteria
History of heart diseases, such as heart failure, angina, and myocardial infarction; History of malignant tumors or pulmonary diseases; History of stroke or ischemic heart diseases; History of taking probiotics or antibiotics within a month; Oral diseases such as untreated oral abscesses or fungal infections; Acute complications of type 2 diabetes mellitus; Presence of problem with taking actigraphy for any reason.

#### 2.2.1 Data collection and analysis

A multi-omics integrative analysis framework was applied. Patients with DKD were first stratified according to phenotypic characteristics. Oral and gut microbial profiles across the three groups were characterized using full-length 16S rRNA gene sequencing. Metabolomic profiling was subsequently performed to identify phenotype-associated metabolic features.

To further evaluate the relationships among microbiota, metabolites, and DKD, weighted gene co-expression network analysis (WGCNA) and integrated multi-omics analyses were conducted. Finally, the potential mediating role of inflammatory cytokines in the association between fecal metabolites and metabolic indicators was examined (**Figure 1b**).

#### 2.2.2 Sample size justification and statistical power

The sample size (n = 535, including n = 347 with complete multi-omics data) was considered adequate based on the following criteria (1): consistency with large-scale metabolic disease studies (27), ensuring sufficient statistical power for microbiome and metabolomics analyses (2); more than 100 participants per subgroup (minimum DKD n = 122), exceeding commonly accepted thresholds for microbiome studies (3); a mediation analysis sample size of n = 347, providing >90% statistical power to detect moderate effects (28) (4); adherence to established ML guidelines (minimum 10 events per feature) combined with cross-validation to reduce overfitting.

#### 2.2.3 Clinical and biochemical indicators

Demographic and clinical data collected included age, sex, body mass index (BMI), waist-to-hip ratio (WHR), and systolic and diastolic blood pressure. Glycemic indicators included fasting blood glucose (FBG), postprandial blood glucose (PBG), and glycated hemoglobin (HbA1c). Lipid and liver function markers included total cholesterol (TC), triglycerides (TG), alanine aminotransferase (ALT), and aspartate aminotransferase (AST). Renal function indicators included serum creatinine (Scr), UACR, and estimated GFR (eGFR). Lifestyle factors—including smoking status (current, former, or never), alcohol consumption (units per week), and specific dietary habits—were recorded to control for potential confounding. Detailed information on medication use in each group was also collected to evaluate potential treatment effects on study outcomes (**Supplementary Table 4**).

#### 2.2.4 Image features of tongue

Tongue images were obtained using the Tongue Diagnosis Analysis System (TFDA-1), developed by the Intelligent Diagnostic Laboratory of Shanghai University of Traditional Chinese Medicine. Images were processed using TDAS V2.0 software (software copyright registration No. 2018SR033451). The TFDA-1 system specifications were as follows: manual mode; shutter speed 1/125; aperture F6.3; ISO 200; correlated color temperature range 4500–7000 K; and illumination intensity 4800 ± 10% lux.

### 2.3 Extraction and analysis of microorganisms

#### 2.3.1 Preparation of oral and fecal samples

Oral microbiota samples were collected from the central region of the tongue dorsum using sterile pharyngeal swabs with at least ten rotational strokes (29). Stool samples were self-collected by participants in the morning using sterile fecal collection devices, targeting the central portion of the specimen (29).

#### 2.3.2 DNA extraction and 16S full-length library construction

All samples were cryopreserved immediately after collection and transported to the laboratory on the same day. Specimens were placed in sterile, enzyme-free Eppendorf tubes, kept on ice, and transferred to a −80 °C freezer within 30 minutes before sequencing. Participants fasted prior to sample collection (29). Bacterial DNA from tongue dorsum swabs and fecal samples was extracted using a swab genomic DNA extraction kit (CW2654, CwBiotech, Beijing, China) and a stool DNA extraction kit (TIANamp Stool DNA Kit, DP328, Tiangen Biotech, Beijing, China), respectively (29). Full-length 16S rRNA gene sequencing (16S-FAST) was performed to achieve species-level taxonomic classification by targeting the nine hypervariable regions and conserved regions of the bacterial 16S rRNA gene (29). Batch effect correction and normalization were conducted to minimize technical bias. Established normalization approaches—such as total sum scaling (TSS) or centered log-ratio (CLR) transformation—were applied as appropriate according to data characteristics. Detailed procedures were described in a previous publication by the research team.

### 2.4 Determination of inflammatory factors in plasma

After an overnight fast of at least 10 hours, venous blood samples were collected in the morning according to the study protocol. Serum samples were obtained by centrifugation and stored at −80 °C until analysis. Inflammatory markers were quantified using enzyme-linked immunosorbent assay (ELISA), including interferon-γ (IFN-γ, MM-0033H1), granulocyte–macrophage colony-stimulating factor (GM-CSF, MM-0037H1), interleukin-6 (IL-6, MM-0049H1), interleukin-4 (IL-4, MM-0051H1), interleukin-10 (IL-10, MM-0066H1), tumor necrosis factor-α (TNF-α, MM-0122H1), interleukin-1β (IL-1β, MM-0181H1), interferon-β (IFN-β, MM-0578H1), lipopolysaccharide (LPS, MM-1309H1), Toll-like receptor 4 (TLR4, MM-13271H1), and transforming growth factor-β (TGF-β, MM-1774H1).

### 2.5 Extraction and determination of metabolites in plasma samples

A total of 347 fasting blood samples (ND = 107, T2DM = 119, DKD = 121) were collected in 5 mL Vacutainer tubes containing ethylenediaminetetraacetic acid (EDTA) as an anticoagulant. Samples were centrifuged at 1,500 × g for 15 minutes at 4 °C.

Liquid chromatography–tandem mass spectrometry (LC–MS/MS) analysis was performed using a Vanquish ultra-high-performance liquid chromatography (UHPLC) system (Thermo Fisher Scientific) equipped with a Waters ACQUITY UPLC BEH Amide column (2.1 mm × 50 mm, 1.7 μm), coupled to an Orbitrap Exploris 120 mass spectrometer (Thermo Fisher Scientific) (30). All differential metabolites identified in this study were annotated at Level 2 (putatively annotated metabolites).

For univariate analysis, metabolite intensities were log10-transformed to approximate normal distribution. Group comparisons were conducted using Student’s t-test. *P*-values were adjusted for multiple testing using the Benjamini–Hochberg (BH) method to control the FDR. For multivariate analysis, variable importance in projection (VIP) scores were derived from orthogonal partial least squares discriminant analysis (OPLS-DA). Metabolites were defined as significantly differential when meeting both criteria: *P* < 0.05 and VIP > 1. Multiple testing correction was consistently applied across omics datasets using the BH procedure.

### 2.6 Co-abundance clustering of metabolites

Co-abundance clusters were constructed based on plasma metabolite profiles using the R package WGCNA (v1.73). The following parameters were applied: soft-thresholding power = 2; minimum module size = 30; merge cut height = 0.25; deepSplit = 2; and partitioning around medoids (PAM) clustering = FALSE. The first principal component (PC1) of each module was calculated using the moduleEigengenes function and used as the representative eigengene value for downstream analyses.

### 2.7 Comparison of KEGG organism genomes/GSEA

Gene Set Enrichment Analysis (GSEA) was performed using the GSEA desktop software with the gene set file cp.kegg.v6.0.symbols.gmt. A nominal *P*-value < 0.05 and an enrichment score (ES) > 1.5 were used as thresholds to identify significantly enriched Kyoto Encyclopedia of Genes and Genomes (KEGG) pathways.

### 2.8 Causal mediation analysis

To evaluate the potential mediating role of inflammatory cytokines in the associations among microbiota, metabolites, and clinical biochemical indicators of T2DM and DKD—including FBG, PBG, HbA1c, Scr, UACR, and eGFR—causal mediation analysis was conducted using the R package mediation (v4.5.0). Two representative microbial taxa (Faecalibacterium and Faecalibaculum) and two key metabolites (mesaconitine and 2-ketobutyric acid) were selected based on their significant associations with the six clinical markers of T2DM and DKD. In addition, inflammatory cytokines showing significant associations with these metabolic and renal indicators were included in the mediation models.

### 2.9 Machine learning methods

Feature selection was performed prior to model construction. A stepwise feature inclusion strategy was applied, whereby features were gradually added to generate multiple candidate subsets. Initially, a small number of features were included, and additional features were incrementally incorporated at each step. For each feature subset, classification models were constructed using random forest algorithms. Model performance was evaluated using k-fold cross-validation (k = 5 or 10), with area under the receiver operating characteristic (ROC) curve (AUC) and accuracy as the primary evaluation metrics. The optimal feature subset for each data modality was determined based on model performance as the number of features increased. To further enhance model robustness and reduce overfitting, a two-step feature selection pipeline was implemented. Model performance was assessed using nested 10-fold cross-validation (nested CV). Specifically, the inner loop performed hyperparameter tuning (e.g., number of trees, maximum depth) through grid search combined with 5-fold cross-validation. The outer loop trained the model using optimized hyperparameters and evaluated performance metrics, including AUC and accuracy. This nested design minimized data leakage between feature selection, hyperparameter optimization, and model evaluation, thereby ensuring unbiased performance estimates. Feature importance was interpreted using SHapley Additive exPlanations (SHAP) values to identify biologically relevant predictors. The final model was trained on the full dataset using the selected features and optimized hyperparameters (31–33). To capture potential non-linear relationships, ML analyses were implemented in Python 3.10.9. Algorithms evaluated included support vector machine (SVM), random forest, adaptive boosting (AdaBoost), XGBoost, gradient boosting decision tree (GBDT), Light Gradient Boosting Machine (LightGBM), categorical boosting (CatBoost), and a voting classifier ensemble. Classification performance metrics were computed using the scikit-learn library (version 1.3.1).

### 2.10 Statistical analysis

Statistical analyses were conducted using SPSS version 25.0 (IBM Corp., Armonk, NY, USA) and R software (version 4.4.3), unless otherwise specified. Normally distributed data were presented as mean ± standard deviation (SD), whereas non-normally distributed variables were reported as median and interquartile range (IQR). Normality was assessed using the Shapiro–Wilk test. Group comparisons were performed using parametric tests for normally distributed variables and non-parametric tests for non-normal data. Specifically, Student’s t-test was used for normally distributed continuous variables, and the Wilcoxon rank-sum test was applied to non-normally distributed variables. Fisher’s exact test was used for categorical variables. For comparisons of microbial taxa or metabolites across the three groups (ND, T2DM, DKD), the Kruskal–Wallis test was performed, followed by Dunn’s *post hoc* test with Bonferroni correction for multiple comparisons. Associations between variables were assessed using Spearman’s rank correlation, with Bonferroni adjustment applied to control for multiple testing. When evaluating adjusted associations, linear mixed-effects models were applied, controlling for age and sex where appropriate. Multiple comparisons were corrected using the Bonferroni method. All statistical tests were two-sided, and *P*-values < 0.05 were considered statistically significant.

## 3 Results

### 3.1 To investigate the association and causal mechanism of multi-site microbiome and metabolites with diabetic kidney disease

**Figure 2:**
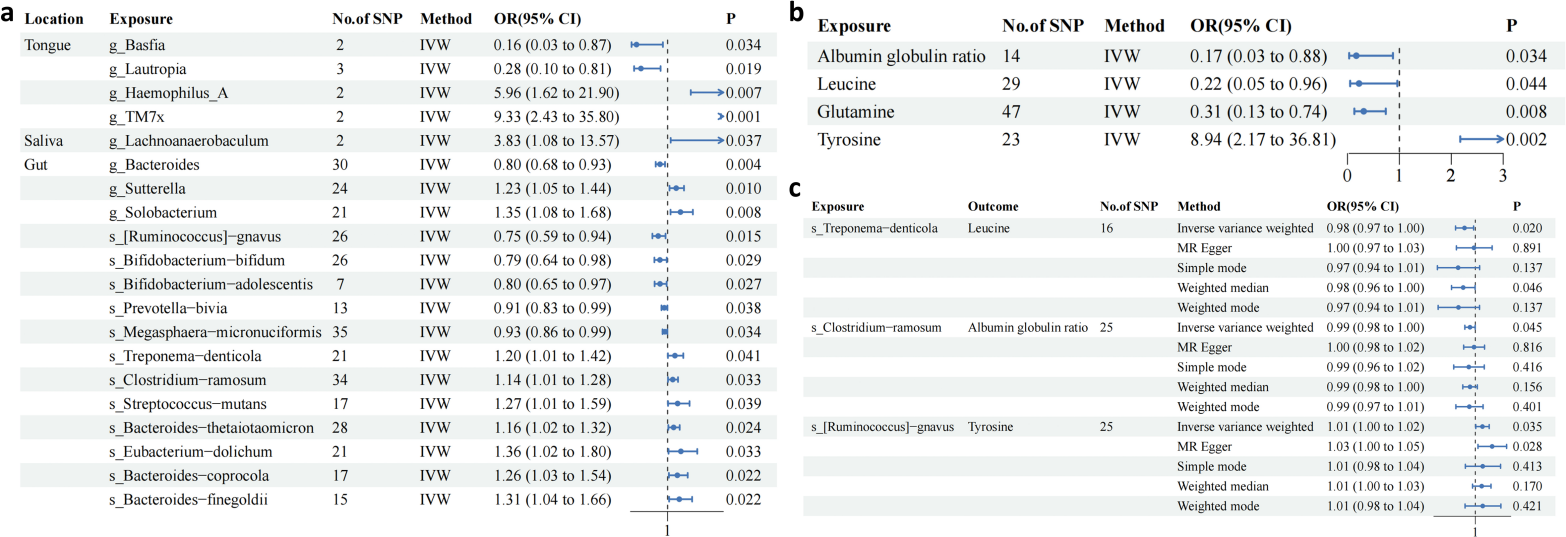
Mendelian randomization analysis: **(a–c)**. Forest plots from Mendelian randomization analysis: **(a)** association between microorganisms and DKD across different sites, **(b)** relationship between metabolites and DKD, **(c)** correlation between positive microorganisms (species level) and positive metabolites.

This study analyzed microbiota from saliva, tongue coating, and gut samples in an East Asian population and identified distinct microbial communities across anatomical sites. All IVs exhibited F-statistics > 10, indicating no evidence of weak instrument bias. Using the IVW method, several microbial genera were significantly associated with DKD risk. Four genera showed positive associations with DKD: Haemophilus-A (OR = 5.96, 95% CI: 1.62–21.90) and TM7x in the tongue coating (OR = 9.33, 95% CI: 2.43–35.80), Lachnoanaerobaculum in saliva (OR = 3.83, 95% CI: 1.08–13.57), and Bacteroides in the gut (OR = 1.27, 95% CI: 1.01–1.59). In contrast, Lautropia (OR = 0.28, 95% CI: 0.10–0.81) and Basfia (OR = 0.16, 95% CI: 0.03–0.87) in the tongue coating, as well as Solobacterium (OR = 0.80, 95% CI: 0.65–0.97) and Sutterella (OR = 0.93, 95% CI: 0.86–0.99) in the gut, were inversely associated with DKD risk (**Figure 2a**; **Supplementary Table 1**). To evaluate the robustness of these findings, additional MR methods—including MR-Egger, weighted median, weighted mode, and simple mode—were applied. The direction and magnitude of associations were generally consistent across methods, supporting the stability of the results (**Figure 2b**; **Supplementary Table 1**). The MR-Egger intercept tests (*P* > 0.05) indicated no evidence of horizontal pleiotropy (**Supplementary Table 2**).

The effects of 27 plasma metabolites on DKD were also examined. Four metabolites were significantly associated with DKD. Higher tyrosine levels were associated with increased DKD risk, whereas glutamine, leucine, and albumin–globulin ratio were inversely associated with disease risk. Sensitivity analyses using MR-Egger, weighted median, weighted mode, and simple mode yielded consistent effect directions (**Figure 2b**; **Supplementary Table 1**). MR-Egger intercept tests (*P* > 0.05) suggested no significant horizontal pleiotropy among the metabolite-related IVs (**Supplementary Table 2**).

Further analyses explored associations between specific microbial taxa and metabolites. Fusobacterium periodonticum in the tongue coating was positively associated with tyrosine levels, whereas Treponema denticola in the gut was inversely associated with leucine. Clostridium ramosum was negatively associated with the albumin–globulin ratio, and Ruminococcus gnavus in the gut was positively correlated with tyrosine-related metabolites (**Figure 2c**; **Supplementary Figure 1a–e**; **Supplementary Table 1**). Although the primary MR analyses identified significant associations among oral and gut microbiota, metabolites, and DKD, formal mediation analysis did not demonstrate significant indirect effects. These findings suggest that the identified metabolites did not statistically mediate the association between microbial alterations and DKD within the current analytical framework. The absence of mediation effects may reflect limited statistical power or measurement variability (**Supplementary Table 1**).

To further evaluate these associations, a clinical cohort study was conducted. The clinical findings were generally consistent with the associations observed in the population-based MR analyses. These results provided additional support for the relationships among microbiota, metabolites, and DKD and informed the design of a subsequent clinical trial.

### 3.2 Phenotypic characteristics of clinical samples

Although MR analysis identified associations among microbiota, metabolites, and DKD, observational data were required to characterize specific microbial and phenotypic markers. Therefore, an observational cohort study including 535 participants (mean age 60.67 ± 0.45 years) was conducted. Biochemical parameters, tongue images, oral microbiota, fecal samples, and serum specimens were collected from all participants. Compared with the ND group (n = 185), the T2DM (n = 228) and DKD groups (n = 122) exhibited significantly higher levels of FBG, PBG, HbA1c, TC, TG, AST, and ALT (all *P* < 0.001). The WHR was significantly higher in the DKD group than in both the ND and T2DM groups. In addition, the prevalence and severity of hypertension were greater in patients with DKD (all *P* < 0.001). Significant differences in Scr, UACR, and eGFR were observed between the DKD and T2DM groups (all *P* < 0.001), whereas other clinical parameters did not differ significantly (all *P* > 0.05; **Supplementary Table 3**).

**Figure 3:**
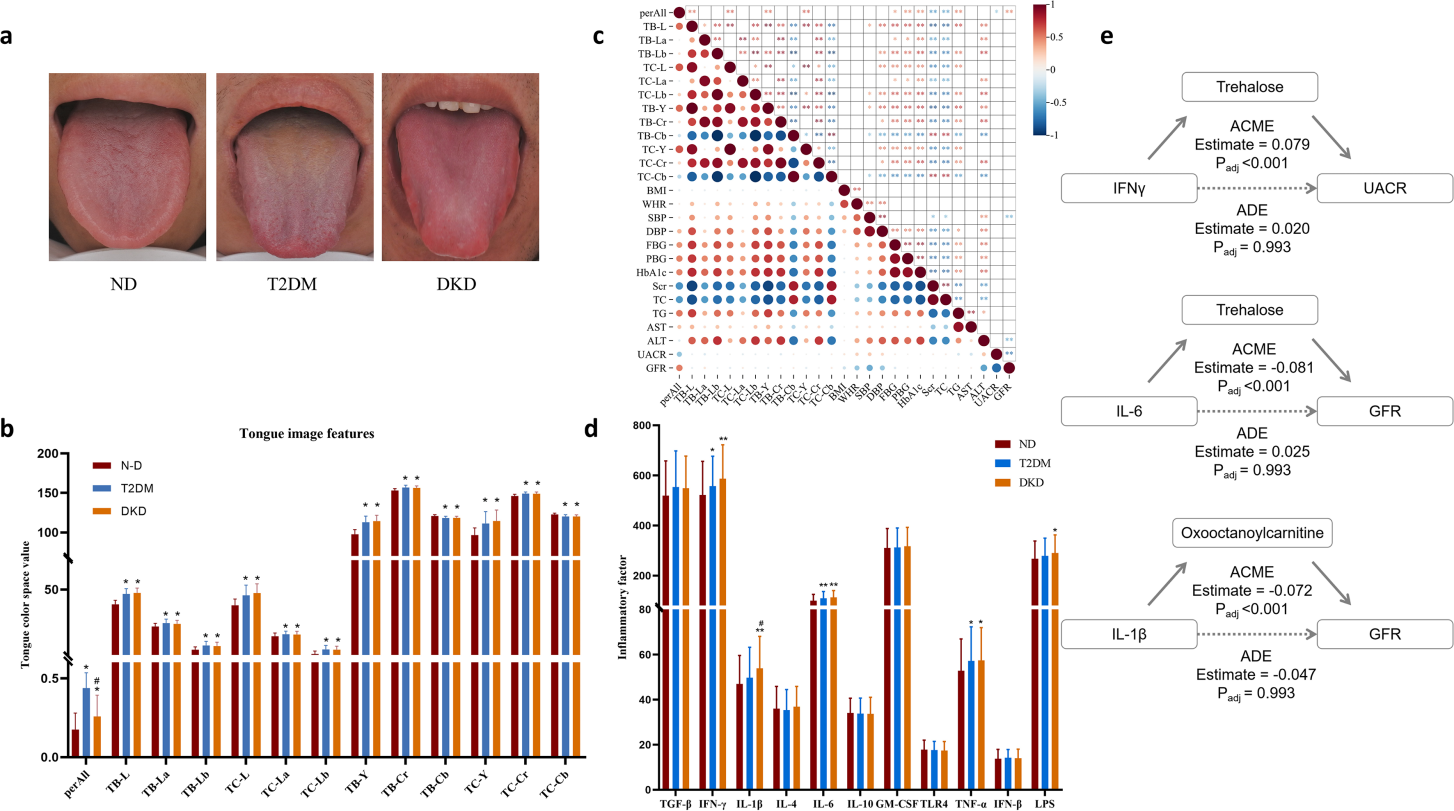
Phenotypic characteristics of clinical samples: **(a)** Standardized tongue images were captured under natural light using the TFDA-1 device. **(b)** After quantifying tongue image features with TDAS V2.0 tongue image analysis software, variations in tongue image parameters among the three groups (ND = 185, T2DM = 228, DKD = 122) were statistically analyzed. Data are presented as mean ± s.e.m. **(c)** Correlation between tongue image parameters and biochemical indicators (**Supplementary Table 3**). **(d)** Changes in serum inflammatory cytokine levels across the three groups (ND = 104, T2DM = 119, DKD = 121) were assessed by ELISA. Data are presented as mean ± s.e.m. **(e)** Representative causal mediation models were used to assess the effect of microbes and metabolites mediating the relationship between inflammatory factors and DKD characteristics in computer simulations. Causal mediation analyses, adjusted for multiple testing, were employed to determine significance. Estimates (β) and adjusted *P*-values (*P*_adj_) of ACME—the indirect effects between metabolites and host markers mediated by metabolites—and average direct effects (ADE)—the direct effects controlling for cytokines—are reported. Detailed information is available in **Supplementary Table 4**. *P<0.05, **P<0.01, ***P<0.001.

Tongue image features (TIFs) differed significantly between the T2DM and DKD groups compared with the ND group. Specifically, CIELAB and YCrCb color-space parameters for the whole tongue region (perALL), tongue body (TB), and tongue coating (TC) were elevated (all *P* < 0.05), consistent with a red tongue body and yellow coating in T2DM. The perALL value was significantly higher in the T2DM group than in the DKD group (*P* < 0.05), suggesting thicker and more greasy tongue coating in T2DM (**Figures 3a, b**). Pearson correlation analysis demonstrated significant associations between tongue image parameters and biochemical indicators. Scr was negatively correlated with TB-L, TB-Y, and perALL, and positively correlated with TB-b and TC-b values. eGFR was positively correlated with perALL. Among glycemic indicators, HbA1c was positively correlated with TB-Y, TB-Cr, and TB-L; PBG was positively correlated with TB-L; and FBG was positively correlated with TC-La (**Figure 3c**; **Supplementary Table 5**).

Inflammatory cytokines IL-6 and TNF-α were significantly elevated in both the T2DM and DKD groups compared with the ND group. IFN-γ and LPS levels were specifically increased in the DKD group (**Figure 3d**). To assess whether inflammatory cytokines mediated the associations between metabolites and DKD-related biochemical markers, causal mediation analysis was performed. For metabolites such as oxooctanoylcarnitine and trehalose, the estimated average causal mediation effects (ACMEs) and corresponding 95% confidence intervals (CIs) varied across models. In analyses examining relationships between inflammatory cytokines (e.g., IL-1β, IL-6, and IFN-γ) and DKD-related indicators (UACR and eGFR), several ACME estimates showed wide CIs, suggesting limited or inconsistent mediation effects. Although some mediation effects remained statistically significant after multiple testing correction, the overall results indicated that the mediating roles of these metabolites were not consistently robust across all examined pathways (**Figure 3e**; **Supplementary Table 6**).

### 3.3 The oral-gut microbiota is reshaped in patients with DKD

**Figure 4:**
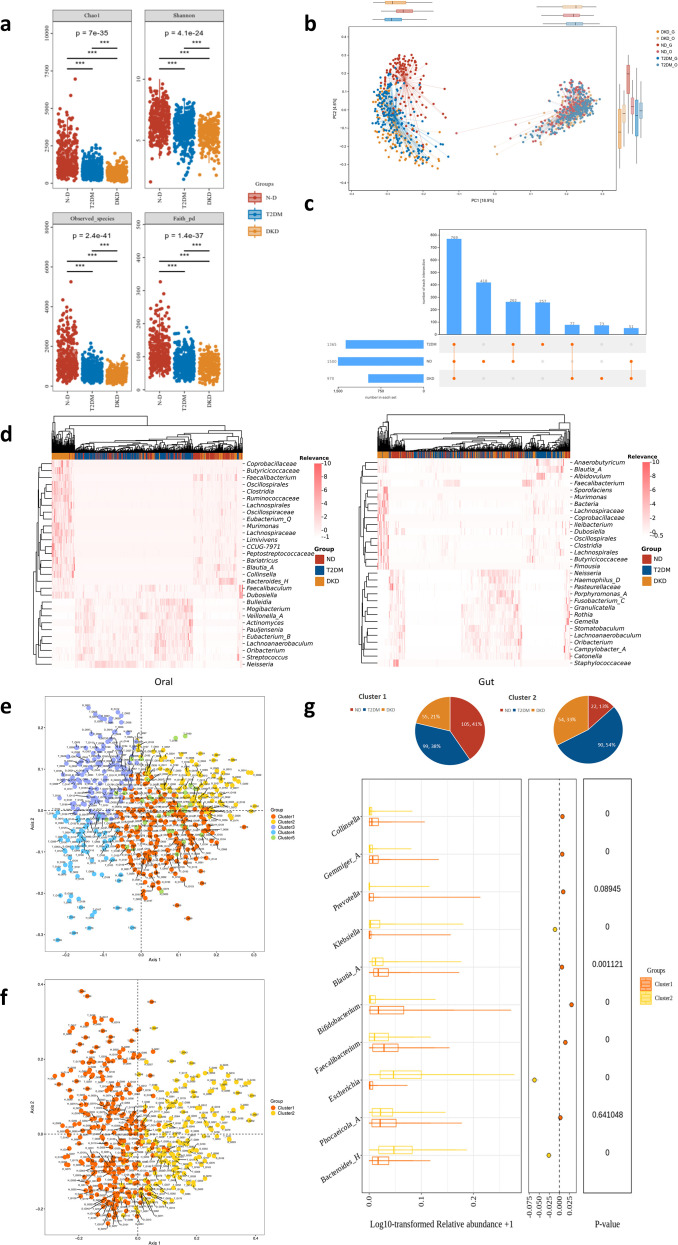
Oral-gut microbiota imbalance in DKD: **(a)** Alpha diversity across the three disease groups (n = 960), showing a significant decrease in diversity with worsening health status (DKD < T2DM < ND, all *P* < 0.01). **(b)** Bray-Curtis differential PCoA plot based on 16S rRNA gene sequencing, highlighting genus-level changes in the oral-gut microbiota across the three disease states (n = 960). Dots represent individual data points, summarized into PCo1 and PCo2. **(c)** Upset diagram showing unique and shared microbial genera across each disease group. **(d)** Heatmap of the top 30 differential marker bacterial genera in both oral and gut microbiota (Oral: n = 535; Gut: n = 425). e-g. Genus-level gut type analysis of oral and gut microbiota using Dirichlet polynomial mixture (DMM) and Jensen-Shannon divergence clustering protocol (PAM-JSD), based on Bray-Curtis (PAM-BC) distances. These analyses examine the microbiota composition in the oral and gut microbiomes (Oral: n = 535; Gut: n = 425). ***P<0.001.

Alpha diversity progressively decreased across disease states, with the lowest diversity observed in the DKD group, followed by the T2DM and ND groups. Differences among the three groups were statistically significant (*P* < 0.01; **Figure 4a**; **Supplementary Table 7**). Beta diversity analysis demonstrated significant differences in microbial community structure across oral and gut microbiota among the three groups. Distinct clustering patterns were also observed within the same anatomical sites across disease states (**Figure 4b**; **Supplementary Figure 2a–c**).

The relative abundance of dominant bacterial taxa differed among groups. The DKD group showed enrichment of genera within the family *Lachnospiraceae*, as well as *Dubosiella* and *Faecalibacterium*. In the oral microbiota, taxa belonging to *Clostridia* were enriched in DKD, whereas *Pauljensenia* was comparatively depleted. In the gut microbiota, *Fusobacterium-C* and members of *Pasteurellaceae* were less abundant in DKD (**Figure 4d**). Enterotype analysis identified five distinct clusters in the oral microbiome. The top ten differentially abundant taxa across clusters were determined, and principal coordinates analysis (PCoA) confirmed significant separation among these groups. In the gut microbiome, two major enterotypes were identified. Cluster 1 was characterized by a high relative abundance of *Faecalibacterium*, whereas cluster 2 was dominated by *Bacteroides-H* (**Figures 4e–g**). Analysis of clinical group distribution within gut enterotypes showed that ND participants were predominantly represented in cluster 1 (41%, n = 105), whereas cluster 2 contained only 13% ND samples (n = 22) (**Figure 4g**). These findings indicated that progression from health to DKD was associated with alterations in digestive tract microbial community structure. The observed shifts may be related to disease-associated inflammatory changes; however, further mechanistic studies are required to clarify the underlying drivers.

**Figure 5:**
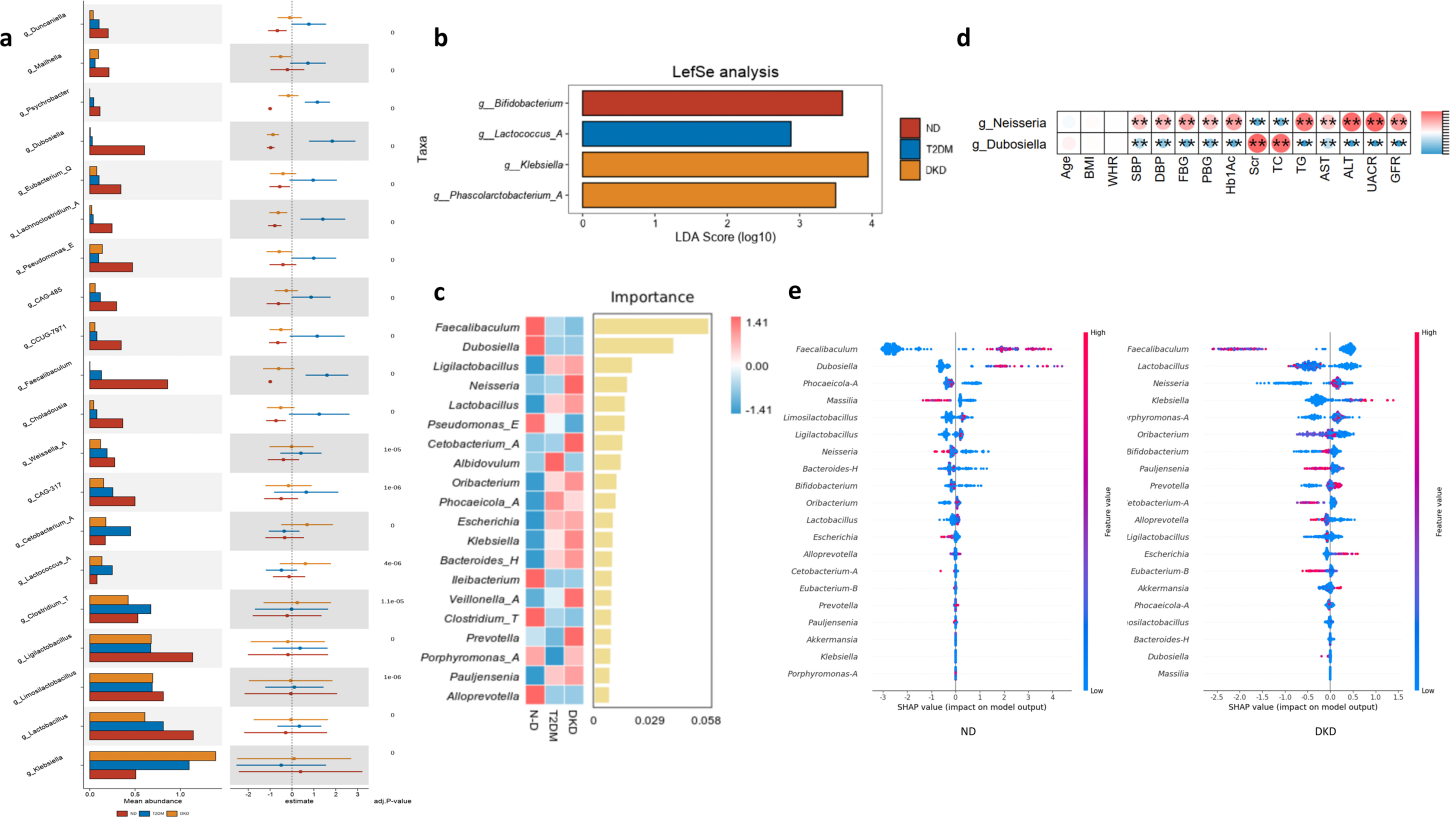
Characteristic markers of DKD: **(a)** The Kruskal-Wallis test was used to compare differences in dominant species at the genus level across the three groups, with *P*-value correction and *post hoc* analysis. The top 20 species with significant abundance differences were selected for illustration (ND = 312; T2DM = 417; DKD = 231). **(b)** The Linear Discriminant Analysis of Effect Size **(LEfSe)** method was employed to analyze dominant species differences at the genus level among the three groups (LDA > 2, *P* < 0.05). The histogram of LDA values for significantly different species highlights those enriched in each group (ND = 312; T2DM = 417; DKD = 231). **(c)** The Random Forest method was used to identify marker species differing at the genus level across the groups. The heatmap shows the abundance distribution of these species, ranked by their importance to the model. **(d)** Heatmap depicting the correlation between gut microbiota and biochemical indicators (*P* < 0.05, R < 0.6). **(e)** SHAP plot visualization of the top 20 genera identified by the XGBoost Regression Model for ND and DKD classification models (ND = 312; DKD = 231). **P<0.01.

Given the substantial alterations in microbial community structure observed in DKD, disease-associated signature taxa were further investigated. Linear discriminant analysis effect size (LEfSe) identified *Klebsiella* as a discriminative biomarker for DKD, with significantly increased abundance across groups (DKD > T2DM > ND; Kruskal–Wallis test, *P* < 0.01). In contrast, *Faecalibaculum* and *Dubosiella* were significantly reduced in DKD (DKD < T2DM < ND; Kruskal–Wallis test, *P* < 0.01; **Figures 5a, b**). ML analyses further highlighted *Faecalibaculum* as a key discriminative taxon across oral and gut microbiota datasets. SHAP analysis demonstrated its strong contribution to model performance, particularly in distinguishing ND samples. Random forest and XGBoost models consistently showed a progressive decrease in *Faecalibaculum* abundance from T2DM to DKD. In contrast, *Dubosiella* exhibited an inverse association with DKD classification probability in the ML models (**Figures 5c, e**). Correlation analyses between microbial taxa and biochemical indicators revealed significant associations for *Neisseria* and *Dubosiella* in the gut microbiota, although their correlation patterns differed. *Dubosiella* was positively correlated with Scr and TC, and negatively correlated with UACR, eGFR, and other metabolic indicators (**Figure 4d**; **Supplementary Figure 2d**). Consistent with the enrichment of DKD samples in cluster heatmap analyses and the increased abundance of *Klebsiella* in gut enterotype cluster 2 (DKD = 54, 33%), these findings suggested that elevated *Klebsiella* and reduced *Faecalibaculum* and *Dubosiella* were associated with DKD status. This pattern was consistent with previous reports.

### 3.4 Microorganism–metabolite relationships are affected in DKD patients

Although the mechanisms by which microbiota alterations influence host physiology remain incompletely understood, metabolic dysregulation is considered a potential pathway. Therefore, untargeted metabolomic profiling was performed on serum samples from study participants.

**Figure 6:**
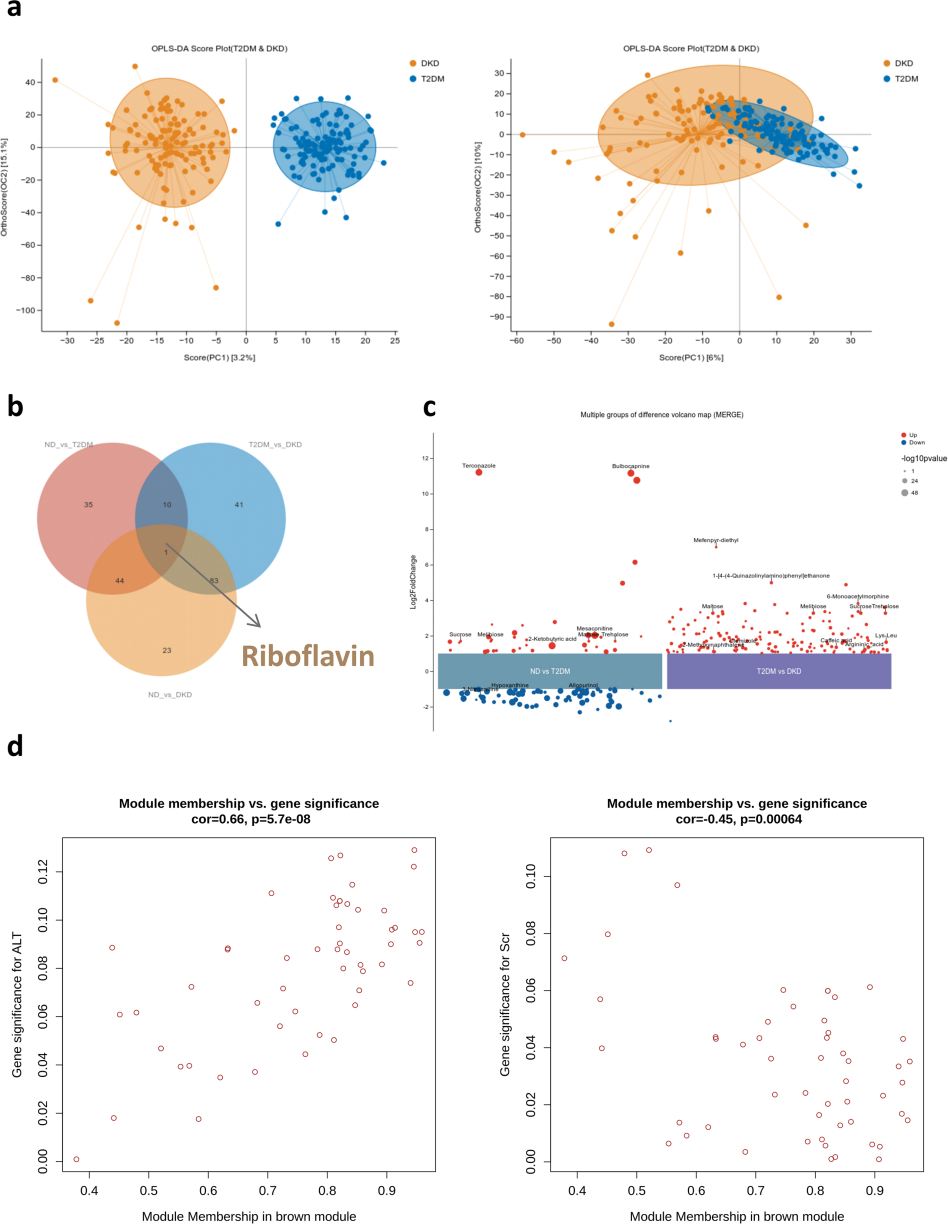
Metabolite alterations in DKD: **(a)** OPLS-DA of untargeted metabolomics analysis in T2DM & DKD, showing POS (left) and NEG (right) metabolites. **(b)** Venn diagram illustrating overlapping differential metabolites in ND & T2DM, ND & DKD, and T2DM & DKD datasets, with Riboflavin identified as a common metabolite. **(c)** Volcano plot showing differential metabolite changes in the ND & T2DM and T2DM & DKD datasets. Red represents FC > 1, *P* < 0.05, and blue represents FC < 1, *P* < 0.05. **(d)** The brown module, containing numerous metabolites, is highlighted; further details are provided in **Supplementary Table 9**.

A total of 404 differential metabolites were identified between the T2DM and DKD groups. OPLS-DA demonstrated clear separation under positive ion mode (POS), whereas partial overlap was observed under negative ion mode (NEG) (**Figure 6a**; **Supplementary Figures 3a, b**,). Riboflavin-related metabolites were differentially expressed during the progression from ND to T2DM and DKD (**Figure 6b**). Untargeted analysis further showed that carbohydrate-related metabolites—including maltose, sucrose, and trehalose—were elevated as T2DM progressed to DKD. Compared with ND and T2DM groups, levels of Lys–Leu, 2-ketobutyric acid, and arginine were also significantly increased in DKD (**Figure 6c**). To investigate host–microbiota metabolic interactions, associations between microbial taxa and metabolites were examined. Given the significant regional differences in oral and gut microbiota, integrated analyses combining oral microbiota, gut microbiota, and serum metabolomic data were conducted across the three groups. Mantel tests demonstrated significant correlations between differential metabolites and both oral and gut microbial communities in T2DM and DKD (all *P* < 0.05; **Supplementary Figure 4b**; **Supplementary Table 8**). WGCNA was performed to evaluate associations among metabolites, clinical biochemical indicators, and disease status. The brown module, enriched in metabolites primarily involved in carbohydrate metabolism—such as 2-hydroxyhexanedioic acid and glucose—showed strong positive correlations with ALT and Scr. This module was also closely associated with DKD-related microbial taxa (**Figure 6d**; **Supplementary Table 9**). These findings suggested coordinated alterations in carbohydrate metabolism and microbial composition in DKD. However, further mechanistic studies are required to determine the causal relationships underlying these associations.

**Figure 7:**
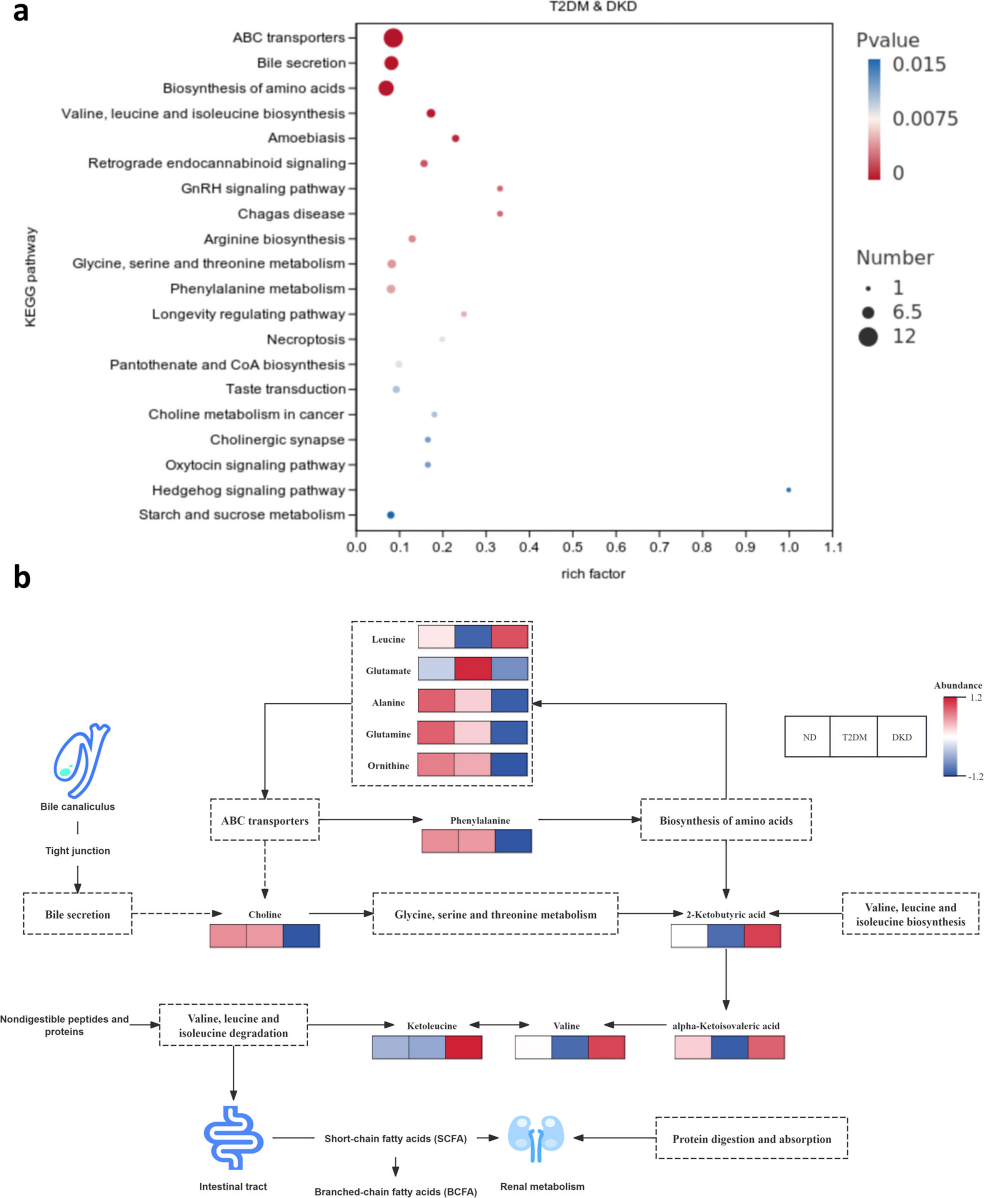
Changes in signaling pathways during disease progression from T2DM to DKD: **(a)** The enrichment factor map displays the top 20 KEGG pathways most significantly enriched with differential metabolites between T2DM and DKD. The horizontal axis represents the enrichment factor for various metabolic pathways, while the vertical axis lists the enriched pathways. Dots indicate the number of metabolites within each pathway, with color corresponding to the FDR value. **(b)** Proposed hypothetical mechanism of the gut-kidney axis in DKD. Metabolic pathways, based on differential metabolites, reveal interrelationships between the degradation and biosynthesis of BCAAs and ABC transporters, with significant changes in ABC transporters across diseases. Pathways, mapped from differential metabolites across the three disease groups, highlight seven primary metabolic pathways. Bile secretion in DKD leads to a decrease in choline, triggering the activation of glycine, serine, and threonine metabolism, and an increase in 2-ketobutyric acid. Additionally, ABC transporters modulate the biosynthesis of amino acid metabolism by inhibiting phenylalanine metabolite production in DKD. These metabolic pathways interact through multiple metabolites. Only leucine metabolites are elevated in DKD, while glutamate increases exclusively in T2DM. Other metabolites, such as alanine, glutamine, and ornithine, show a decreasing trend. The biosynthesis of amino acids also influences 2-ketobutyric acid. Furthermore, BCAAs biosynthesis is closely linked to their degradation via 2-ketobutyric acid. Both alpha-ketoisovaleric acid and valine signaling pathways contribute to ketoleucine in DKD. Through multiple signaling pathways, metabolite alterations influence the conversion of short-chain fatty acids (SCFA) to branched-chain fatty acids (BCFA), impacting renal metabolism via protein digestion and absorption signaling pathways.

Given the coordinated alterations observed in the gut microbiota and metabolome in DKD, this study examined whether metabolic pathway changes reflected shifts in microbial functional potential. Pathway enrichment analyses indicated that the biosynthesis and degradation of branched-chain amino acids (BCAAs; valine, leucine, and isoleucine) were significantly associated with the progression from T2DM to DKD (KEGG: FDR = 0.000, *P* < 0.001; GSEA: normalized enrichment score [NES] = −1.766, *P* < 0.05; **Supplementary Figure 5b**). Alterations in these BCAA-related pathways were significantly correlated with specific microbial taxa, including *Klebsiella* and *Fusobacterium-C*, as well as with the ATP-binding cassette (ABC) transporter pathway (**Figure 7a**; **Supplementary Figure 7a, b**; **Supplementary Table 10**). ABC transporters were significantly activated in both T2DM and DKD (GSEA: NES = 1.550, *P* < 0.001), while taurine and hypotaurine metabolism was notably inhibited (NES = 1.490, *P* < 0.01, **Supplementary Figure 5c**). These findings suggest that the loss of beneficial bacteria, such as *Faecalibaculum* and *Dubosiella*, in the oral and gut microbiota may disrupt metabolic processes, particularly the degradation and biosynthesis of leucine, isoleucine, and valine (**Supplementary Figure 5c**). This disruption can alter the metabolite pool (e.g., ATP levels and phenolic content) and impair cellular homeostasis. Furthermore, these metabolic alterations may induce abnormal bile acid function, promote the production of inflammatory factors, and ultimately contribute to the development of DKD (**Figure 7b**).

### 3.5 Machine learning can effectively distinguish DKD from T2DM through microbiota-based biomarkers

**Figure 8:**
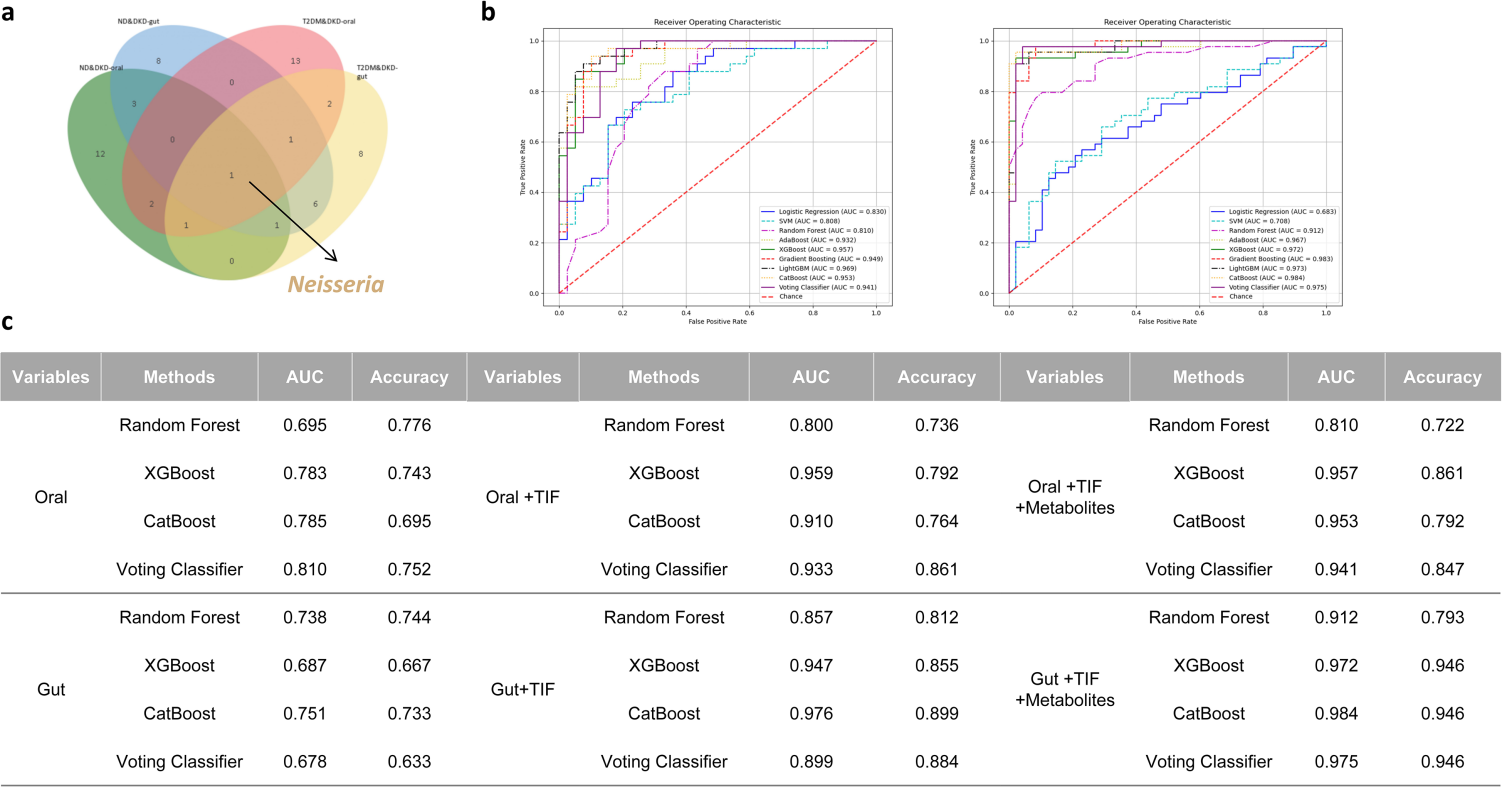
Feature selection and model prediction of ML methods to differentiate patients with DKD and those with T2DM: **(a)** The four panels display the top features selected by CatBoost. A Venn diagram illustrates the overlap and uniqueness of features between the oral and gut microbiota for ND & DKD and T2DM & DKD. **(b)** Construction of a syndrome classification model using oral (left)/gut (right) microbiota, tongue image features, and metabolites, along with the corresponding Receiver Operating Characteristic (ROC) curve. **(c)** Comparison of diagnostic efficiency across different ML methods.

To identify features distinguishing T2DM from DKD, the dataset was divided into a training set (30%) and a validation set (70%) for ML analyses (**Figure 1c**). Classification models were constructed to evaluate the diagnostic potential of microbiota-based multi-omics biomarkers for DKD. The predictive performance of individual omics datasets was first assessed using ROC curves and corresponding AUC values generated by a random forest classifier. Candidate features were derived from TIFs, microbiota profiles, and metabolomic data. Feature selection identified seven informative TIF variables, three metabolite features, and nineteen microbial taxa that discriminated between T2DM and DKD (**Supplementary Figure 8**; **Supplementary Table 11**). Based on these selected features, three independent diagnostic models were constructed. In the microbiota-only models, oral microbiota demonstrated higher diagnostic performance (accuracy = 0.752) compared with gut microbiota (accuracy = 0.633). Among the evaluated algorithms, CatBoost achieved the best performance (oral AUC = 0.785; gut AUC = 0.751). In models distinguishing ND & DKD and T2DM & DKD groups, Neisseria ranked among the top 20 microbial features (**Figure 8a**; **Supplementary Table 12**). Integration of TIFs substantially improved model performance. The combined oral microbiota + TIF model achieved an accuracy of 0.861, with XGBoost yielding the highest AUC (0.959). The gut microbiota + TIF model reached an accuracy of 0.884. Further incorporation of metabolomic features enhanced predictive performance. The oral microbiota + TIF + metabolite model achieved an accuracy of 0.861 using XGBoost, while the gut microbiota + TIF + metabolite model reached an accuracy of 0.946 (**Figures 8b, c**).

## 4 Discussion

DKD is a major microvascular complication of T2DM, yet its etiology and underlying mechanisms remain incompletely understood. To better characterize host–microbiota interactions in DKD, this study conducted a comprehensive multi-modal investigation integrating MR, microbiome profiling, metabolomics, and ML analyses across oral and gut sites. Accumulating evidence indicates that the gut microbiota contributes to the pathogenesis of diabetes and its complications (34–37). In contrast, the oral microbiota—despite representing the initial segment of the digestive tract—has been comparatively underexplored in metabolic disease research. Moreover, mechanistic insights in humans remain limited, partly due to insufficient integration of metabolomic data. A major strength of the present study was the application of MR analysis to evaluate potential associations between microbiota from different anatomical sites and circulating metabolites in DKD, followed by validation in a well-characterized clinical cohort combining oral and gut microbiota profiling with serum metabolomics.

Although MR analysis suggested potential causal relationships, the clinical data were observational and therefore cannot establish definitive causality. Nevertheless, the integrative multi-omics framework enabled identification of coordinated alterations in microbiota and metabolites associated with DKD. These findings underscore the importance of examining microbial–metabolic interactions across multiple anatomical niches. Microbial communities from upstream (oral) and downstream (gut) regions of the GI tract exhibited distinct characteristics and were associated with DKD status, supporting their potential value as complementary biomarkers.

Previous studies have reported associations between oral microbial dysbiosis and systemic diseases (38, 39), with immune and inflammatory pathways proposed as mediating mechanisms (40, 41). In the present study, both oral and gut microbiota exhibited significant alterations in DKD. Specifically, *Faecalibaculum* and *Dubosiella* were reduced in DKD compared with ND and T2DM groups, whereas *Klebsiella* was enriched. These findings are consistent with prior reports describing increased *Klebsiella* abundance in DKD (42–44). *Klebsiella* is a normal inhabitant of the GI tract microbiome in healthy humans and animals but can cause extraintestinal infections, including urinary tract infections, pneumonia, and septicemia (45, 46).

This study emphasizes the advantages of a comprehensive omics approach for investigating the role of microbes and metabolites in the pathogenesis of DKD. Riboflavin metabolites were identified as differentially expressed during the progression from ND to T2DM and DKD. Deficiencies in several B vitamins, including B1 (thiamine), B2 (riboflavin), B3 (niacin/nicotinamide), B5 (pantothenic acid), B6 (pyridoxine), B8 (biotin), B9 (folate), and B12 (cobalamin), have been shown to influence the development of CKD, diabetes, and DKD (47, 48). Additionally, biomarkers linked to DKD-related metabolic processes, such as fatty acids and amino acids (e.g., N-Acetyl-D-galactosamine 4-sulfate, 2-Hydroxyhexanedioic acid, Lys-Leu, 2-Ketobutyric acid, and Arginine), were identified. These metabolic biomarkers play critical physiological roles and are involved in multiple metabolic pathways. Moreover, antidiabetic and antihypertensive drugs can disrupt microbiota and metabolite profiles via multiple mechanisms. These drugs may directly alter gut microbial composition by either inhibiting or promoting the growth of specific bacteria, thereby disrupting microbiome balance (15, 49, 50). While our study identifies correlations between microbial genera and metabolic pathways, further functional studies are necessary to clarify the underlying mechanistic links.

Differences in metabolites highlighted by multi-omics changes may reflect microbial gene expression. In the current study, a significant accumulation of monosaccharides and fatty acids was observed in DKD patients. Tadashi Takeuchi et al. demonstrated that *in vivo* administration of *A. indistinctus* improved lipid accumulation, alleviated insulin resistance, and reduced intestinal monosaccharide levels (51). These findings underscore the importance of understanding the mechanisms driving glycogen accumulation and disrupted fatty acid metabolism. Specifically, in the oral cavity, the genus *Desulfovibrio-R* decreased in DKD and was positively correlated with Oxooctanoylcarnitine. In the gut, *Pseudomonas-E* also decreased and exhibited a positive correlation with Oxooctanoylcarnitine, while Murimonas increased. Some metabolites, such as D-Ribose, were negatively correlated with Murimonas. Oxooctanoylcarnitine, an acylcarnitine, is an intermediate product of fatty acid metabolism (52–54). As a metabolomics biomarker, it reflects abnormal energy metabolism.

Although mediation analysis did not support a significant indirect effect of metabolites in the MR framework, this result does not exclude biologically meaningful interactions. Gut microbes influence host physiology through multiple mechanisms beyond metabolite production, including modulation of immune cell function via pattern-recognition receptors, alteration of intestinal barrier integrity, and systemic inflammatory activation (16, 55). Therefore, the association between microbiota and DKD may also involve non-metabolic pathways that were not directly evaluated in this study. In addition, elevated circulating metabolites in DKD may not solely reflect increased microbial production. Progressive renal dysfunction can impair metabolite clearance, leading to systemic accumulation. Declining eGFR and tubular dysfunction may contribute to reduced excretion of metabolic byproducts. For example, Ahmed et al. reported accumulation of protein-bound uremic toxins, including p-cresyl sulfate (PCS) and indoxyl sulfate (IS), in patients with CKD due to impaired renal clearance (56). Thus, both altered microbial metabolism and reduced renal elimination may underlie the observed metabolomic patterns.

Pathway enrichment analyses using KEGG and GSEA highlighted the involvement of BCAA metabolism. Activation of ABC transporters and alterations in taurine and hypotaurine metabolism were also observed during disease progression. BCAAs play essential roles in metabolic homeostasis and have been extensively studied in obesity, diabetes, and cardiovascular disease (57, 58). In this study, microbial taxa such as *Klebsiella* were associated with ABC transporter and BCAA-related pathways. These coordinated shifts suggest that microbial alterations may be linked to pathway-level metabolic reprogramming; however, direct functional validation is required.

ML approaches were further applied to evaluate the diagnostic potential of microbiota, metabolites, and TIFs. Microbial taxa, including *Neisseria*, contributed substantially to model performance in distinguishing ND, T2DM, and DKD groups. Integration of multi-omics data improved classification accuracy, exceeding 90% in combined models. These findings indicate that multi-layered biomarker integration may enhance risk stratification for DKD. ML techniques are increasingly utilized to identify biomarkers and risk predictors in DKD and other metabolic diseases (59–61). By incorporating microbiota from both oral and intestinal sites, together with metabolomic and phenotypic data, the present study provides a framework for non-invasive DKD risk assessment. Nevertheless, prospective validation in independent cohorts is required before clinical implementation.

This study has several important limitations. First, the absence of an external validation cohort limits the generalizability of the findings. Although internal validation procedures, including cross-validation, were performed, prospective evaluation in independent multi-center cohorts is necessary to confirm the robustness and reproducibility of the predictive models. Second, the clinical analyses were observational in nature. While MR suggested potential causal relationships, these findings should be interpreted cautiously. Formal mediation analyses did not yield statistically significant indirect effects. This may partly reflect limited statistical power, particularly given the relatively modest sample size of the metabolic GWAS datasets and the small effect sizes typically observed for individual metabolites in two-sample MR analyses. Third, although the microbiota and metabolomic patterns observed in this cohort were generally consistent with previous reports, the results may not be universally applicable. Disease distribution and microbial composition can be influenced by demographic factors such as age and sex. Despite efforts to control for confounders, residual bias—particularly related to sex imbalance—cannot be excluded. In addition, although medication use was recorded, strict exclusion based on pharmacological treatment was not feasible due to practical considerations. Antidiabetic and antihypertensive therapies may influence microbiota composition and metabolite profiles, thereby affecting study outcomes. Future investigations should incorporate more rigorous medication stratification criteria, larger sample sizes, and longer follow-up durations to improve internal validity. Furthermore, functional predictions were based on full-length 16S rRNA gene sequencing combined with PICRUSt2 analysis. While informative, this approach infers microbial function indirectly. Metagenomic sequencing, metatranscriptomics, or fecal metabolomics will be required in future studies to directly characterize microbial functional capacity and host–microbe metabolic interactions. Finally, the study was conducted at a single center. External validation across multiple institutions and diverse populations will be essential to evaluate model performance across different clinical settings. Prospective studies assessing clinical utility—including integration into diagnostic workflows and evaluation of patient outcomes—are also warranted.

This experimental study demonstrated significant differences in the microbial profiles of the oral cavity and intestines in patients with DKD, emphasizing the importance of considering regional variations when screening for biomarkers and diagnosing the disease. Notably, the detection of *Klebsiella* and *Faecalibaculum* abundance in the digestive tract of patients presents an opportunity for early screening of DKD. Through multi-omics analysis combining microbiota and metabolites from different regions of DKD patients, specific and unique metabolic pathways related to BCAA and ATP were found to correlate with microbiota changes in DKD. Our findings highlight the potential of microbiota, metabolites, and TIFs as biomarkers for diagnosing DKD. The integration of multi-omics data with machine learning presents a promising approach for the non-invasive detection of early DKD and the development of personalized treatment strategies.

## Data Availability

The datasets presented in this study can be found in online repositories. The names of the repository/repositories and accession number(s) can be found in the article/**Supplementary Material**.

## Supplementary material

Supplementary Figure 1 Positive microorganisms & Positive metabolites Mendelian randomization analysis: a-c.Scatter plots of different MR Methods (inverse variance weighting, MR-Egger, weighted median, simple mode, and weighted mode), where the horizontal axis represents the effect of SNPS on the microbiota and the vertical axis represents the effect of SNPS on metabolites. a)*Clostridium ramosum* was negatively associated with the Albumin-globulin ratio, b)*Treponema denticola* was negatively correlated with Leucine, c) *Ruminococcus gnavus* was positively correlated with Tyrosine. d-f. Forest plots for each SNP locus showing the effect estimates for the individual SNPS and their 95% confidence intervals, and the overall effect estimates by the combined MR Egger and inverse variance weighting method for all SNPS are summarized at the bottom. d)*Clostridium ramosum* was negatively associated with the Albumin-globulin ratio, e)*Treponema denticola* was negatively correlated with Leucine, f) *Ruminococcus gnavus* was positively correlated with Tyrosine.

Supplementary Figure 2 Comprehensive analysis of microbiome network structure, disease state association and clinical parameter correlation: a. The microbial co-occurrence network diagram reflects the interaction between the species in the microbiome. It was divided into two modules. The nodes in the graph represent different microbial species, and the size of the node reflects its degree in the network, which is the number of connections with other microbial species. The color of the nodes is used to distinguish different microbial taxa. The edges (i.e., the lines between nodes) represent the correlation between microorganisms, and their color and width represent the positive and negative direction and strength of the correlation, respectively. b. Bar graphs compare the abundance and number of microbial taxa under different disease states (ND, TZDM, DKD). The high degree of the bar graph visually demonstrates the differences in the number of microorganisms in each disease state. c. Boxplot, visually showing the difference of beta diversity between oral and intestinal tract in different disease states (ND, TZDM, DKD), Kruskal-Wallis rank sum test was used for difference test, and dunn's test was used as post hoc test to test the significance of difference between each two groups. d. Association analysis plots showing the correlations between oral microbial species and various clinical parameters. Color and magnitude indicate the strength and direction of the correlation, and asterisks indicate the level of significance. The legend on the right explains the color coding and salience markers. The oral microbiota was correlated with ALT, UACR and GFR (*P*<0.05).

Supplementary Figure 3 Multivariate statistical OPLS-DA analysis for pairwise comparisons of metabolites: a. Permutation test plots of T2DM&DKD showing POS (left) and NEG (right) metabolites. b. OPLS-DA of ND&T2DM analyzed by untargeted metabolomics shows POS (left) and NEG (right) metabolites.

Supplementary Figure 4 Metabolite alterations in DKD: a. Differential marker metabolites between T2DM and DKD identified using the support vector machine (SVM) model; b. The Mantel test was employed to assess correlations between the top 20 most abundant genera across the three groups, analyzing the relationship between these factors and community composition, as well as their association with tongue objectivization, biochemical, and metabolite indices.

Supplementary Figure 5 Multimodal analysis reveals the interaction between the microbiome and the metabolome under healthy and diseased conditions: **(a)** Linear correlation scatter plots showed the correlation between oral microbiota (*Desulfovibrio-R*) and metabolites(Oxooctanoylcarnitine) in the difference between T2DM and DKD(left)and the correlation between gut microbiota (*Murimonas*) and metabolites(D-Ribose) in the difference between T2DM and DKD(right). **(b)** The network diagram is constructed based on the correlation between metabolic pathways and microbial functions. The circles represent a specific metabolic pathway or microbial function. The color and category of the circles indicate the name of the pathway or function they represent. The size of the circles reflects the connectivity or importance of the pathway or function in the network. The lines between the circles represent the interaction relationship between the metabolic pathway and microbial function. The thickness of the lines may reflect the intensity or reliability of the interaction. **(c)** The bar chart shows the relative abundance of the top 20 microbial genera involved in the degradation of valine, leucine, and isoleucine in different experimental groups.

Supplementary Figure 6 Analysis of metabolic pathway differences and functions among different health states: a. The bar charts of KEGG metabolic pathway enrichment analysis respectively show the enrichment of differentially regulated metabolic pathways among ND&T2DM, ND&DKD, and T2DM&DKD. b. The enrichment factor map shows the top 20 KEGG pathways that are most significantly enriched with differential metabolites between ND and T2DM. c. The GSEA plot shows the enrichment of ABC transporter-related gene sets (on the left) and the gene sets related to the degradation metabolism pathway of valine, leucine, and isoleucine (on the right) in the ordered dataset. The enrichment trend is displayed through the Enrichment Score (ES) curve.

Supplementary Figure 7 Metabolite alterations in DKD: **(a)** Differential marker metabolites between T2DM and DKD identified using the support vector machine (SVM) model; **(b)** The Mantel test was employed to assess correlations between the top 20 most abundant genera across the three groups, analyzing the relationship between these factors and community composition, as well as their association with tongue objectivization, biochemical, and metabolite indices.

Supplementary Figure 8 The performance of different omics data in the model: The line graph presents the performance evaluation results of the model constructed based on Tongue image features, Metabolomic and Microbiota data. Each subgraph corresponds to a different data type and shows the trend of the AUC (Area Under the Curve) score and accuracy score of the model on that data as the number of included features changes. a) Tongue image features, b) Metabolomic, c) Microbiota.

Supplementary Table 1 MR result.

Supplementary Table 2 Sensitive analysis of the association between DKD and identified bacterial traits.

Supplementary Table 3 Clinical characteristics of study participants.

Supplementary Table 4 Medication Usage Across Groups.

Supplementary Table 5 Correlation analysis of tongue body characteristics and biochemical indicators(Pearson).

Supplementary Table 6 Causal mediation analysis between Microorganisms & metabolites, plasma cytokines, and clinical markers.

Supplementary Table 7 Alpha diversity between groups.

Supplementary Table 8 Analysis of the correlation between the microbial community and metabolites.

Supplementary Table 9 The correlation between the brown module of WGCNA and the biochemical indicators.

Supplementary Table 10 KEGG differential substance enrichment analysis.

Supplementary Table 11 The dataset included in the machine learning model.

Supplementary Table 12 The top 20 microorganisms in pairwise comparisons under the CatBoost model.
