# Supplementary material for: Integrated multi-omics analysis unveils microbiota-metabolite-host interactions and novel biomarkers for early diabetic kidney disease diagnosis
Source: Front Immunol. 2026 Mar 9;17:1781013. doi: 10.3389/fimmu.2026.1781013 (PMC13006260; doi:10.3389/fimmu.2026.1781013)
Supplement: Supplementary Figure 1 — Positive microorganisms & Positive metabolites Mendelian randomization analysis: a-c.Scatter plots of different MR Methods (inverse variance weighting, MR-Egger, weighted median, simple mode, and weighted mode), where the horizontal axis represents the effect of SNPS on the microbiota and the vertical axis represents the effect of SNPS on metabolites. a)Clostridium ramosum was negatively associated with the Albumin-globulin ratio, b)Treponema denticola was negatively correlated with Leucine, c) Ruminococcus gnavus was positively correlated with Tyrosine. d-f. Forest plots for each SNP locus showing the effect estimates for the individual SNPS and their 95% confidence intervals, and the overall effect estimates by the combined MR Egger and inverse variance weighting method for all SNPS are summarized at the bottom. d)Clostridium ramosum was negatively associated with the Albumin-globulin ratio, e)Treponema denticola was negatively correlated with Leucine, f) Ruminococcus gnavus was positively correlated with Tyrosine. [file Table1.docx]

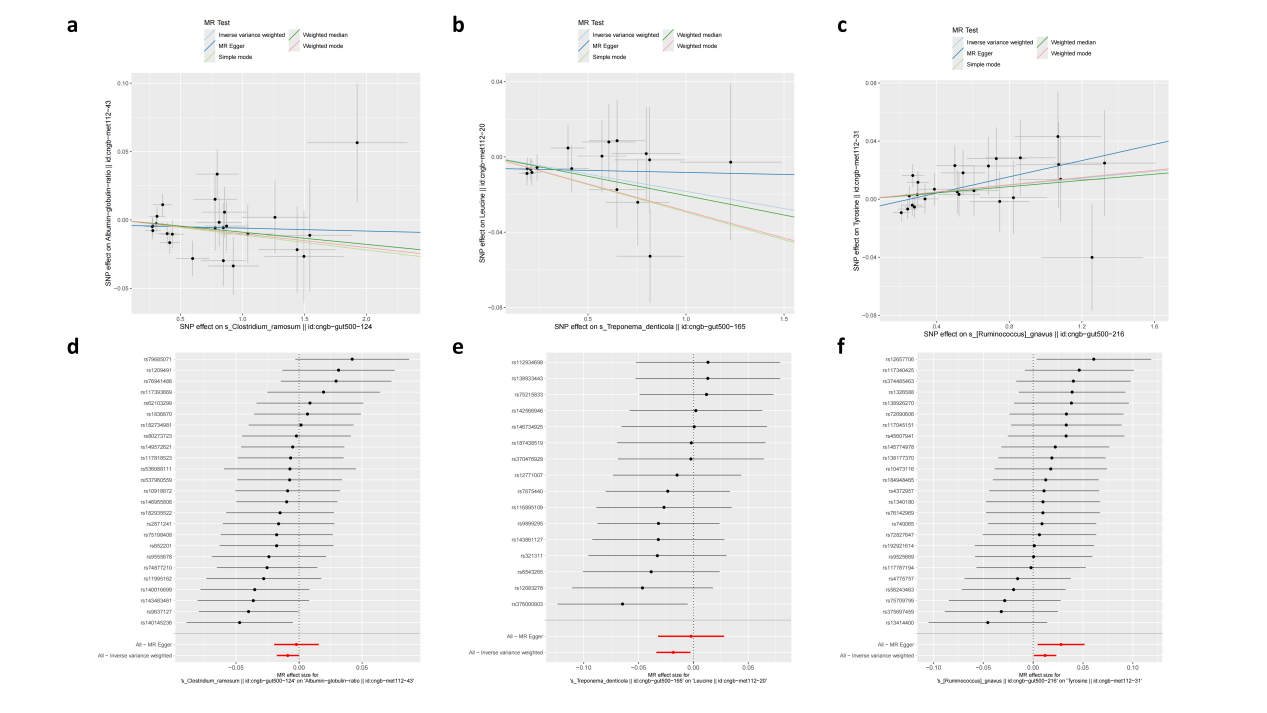


**Supplementary** **Fig. 1 | Positive microorganisms & Positive metabolites Mendelian randomization analysis:** a-c.Scatter plots of different MR Methods (inverse variance weighting, MR-Egger, weighted median, simple mode, and weighted mode), where the horizontal axis represents the effect of SNPS on the microbiota and the vertical axis represents the effect of SNPS on metabolites. a)*Clostridium ramosum* was negatively associated with the Albumin-globulin ratio, b)*Treponema denticola* was negatively correlated with Leucine, c) *Ruminococcus gnavus* was positively correlated with Tyrosine. d-f. Forest plots for each SNP locus showing the effect estimates for the individual SNPS and their 95% confidence intervals, and the overall effect estimates by the combined MR Egger and inverse variance weighting method for all SNPS are summarized at the bottom. d)*Clostridium ramosum* was negatively associated with the Albumin-globulin ratio, e)*Treponema denticola* was negatively correlated with Leucine, f) *Ruminococcus gnavus* was positively correlated with Tyrosine.

**
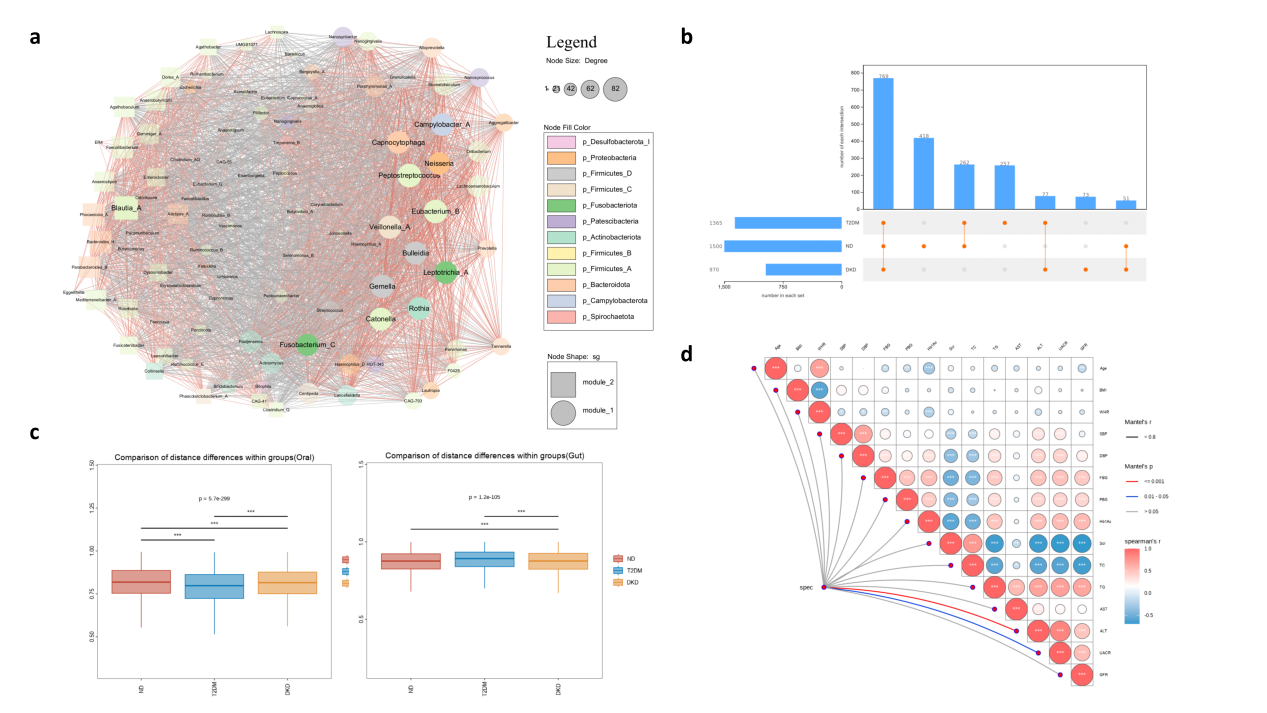
**

**Supplementary** **Fig. 2 | Comprehensive analysis of microbiome network structure, disease state association and clinical parameter correlation:** a. The microbial co-occurrence network diagram reflects the interaction between the species in the microbiome. It was divided into two modules. The nodes in the graph represent different microbial species, and the size of the node reflects its degree in the network, which is the number of connections with other microbial species. The color of the nodes is used to distinguish different microbial taxa. The edges (i.e., the lines between nodes) represent the correlation between microorganisms, and their color and width represent the positive and negative direction and strength of the correlation, respectively. b. Bar graphs compare the abundance and number of microbial taxa under different disease states (ND, TZDM, DKD). The high degree of the bar graph visually demonstrates the differences in the number of microorganisms in each disease state. c. Boxplot, visually showing the difference of beta diversity between oral and intestinal tract in different disease states (ND, TZDM, DKD), Kruskal-Wallis rank sum test was used for difference test, and dunn's test was used as post hoc test to test the significance of difference between each two groups. d. Association analysis plots showing the correlations between oral microbial species and various clinical parameters. Color and magnitude indicate the strength and direction of the correlation, and asterisks indicate the level of significance. The legend on the right explains the color coding and salience markers. The oral microbiota was correlated with ALT, UACR and GFR (*P*<0.05).


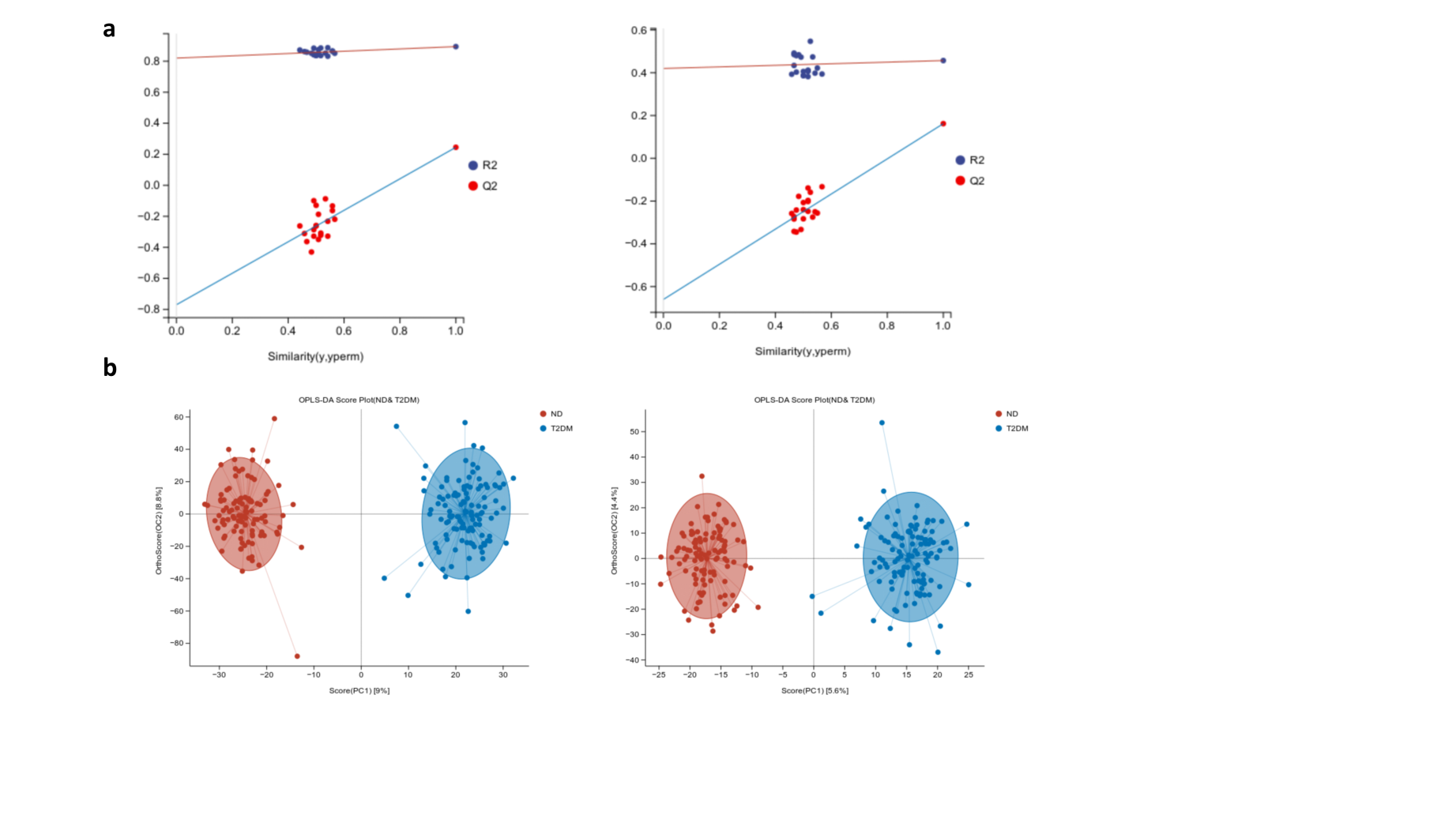


**Supplementary** **Fig. 3 | Multivariate statistical OPLS-DA analysis for pairwise comparisons of metabolites:** a. Permutation test plots of T2DM&DKD showing POS (left) and NEG (right) metabolites. b. OPLS-DA of ND&T2DM analyzed by untargeted metabolomics shows POS (left) and NEG (right) metabolites


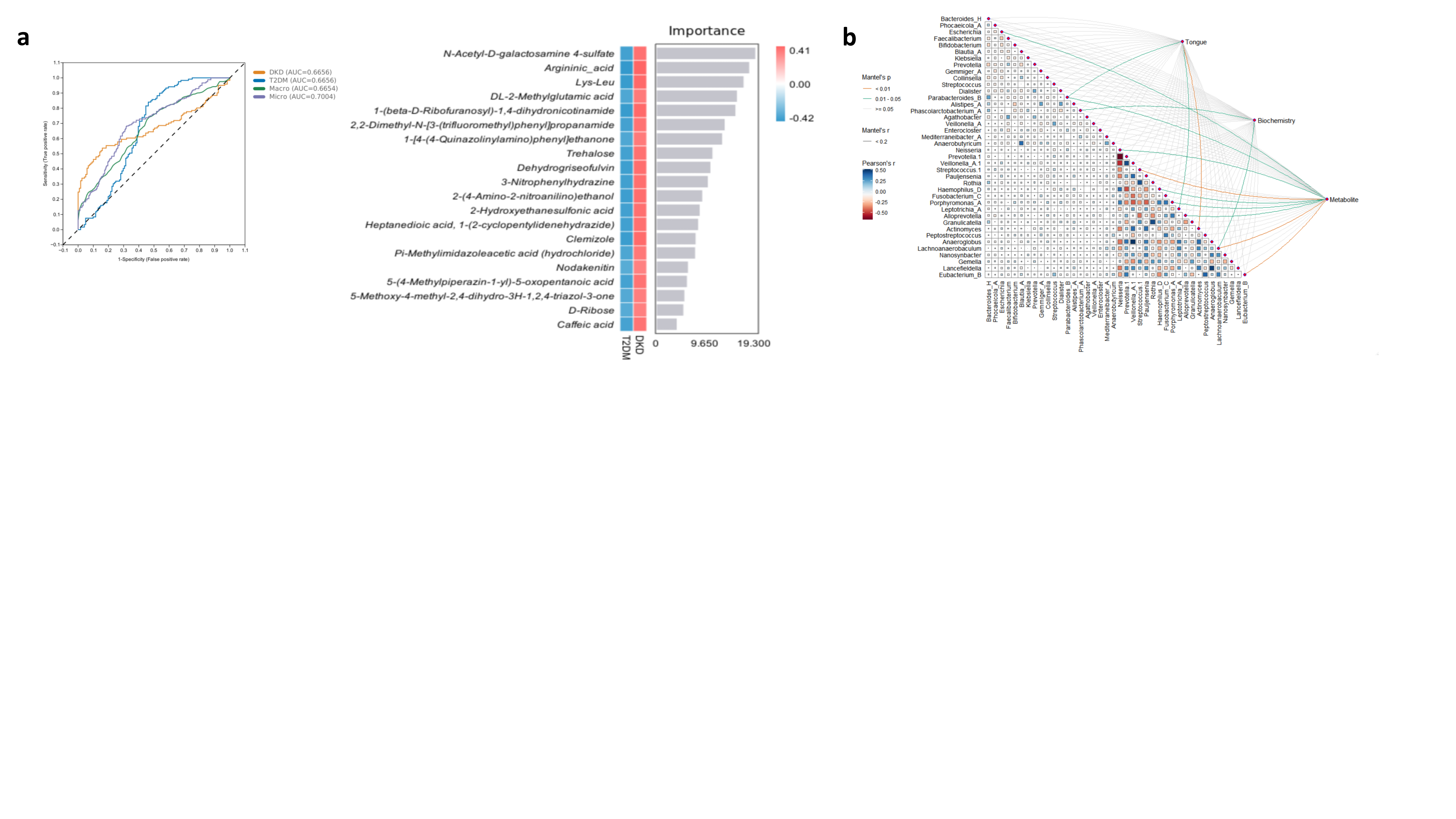


**Supplementary** **Fig. 4 | Metabolite alterations in DKD:** a. Differential marker metabolites between T2DM and DKD identified using the support vector machine (SVM) model; b. The Mantel test was employed to assess correlations between the top 20 most abundant genera across the three groups, analyzing the relationship between these factors and community composition, as well as their association with tongue objectivization, biochemical, and metabolite indices.

**
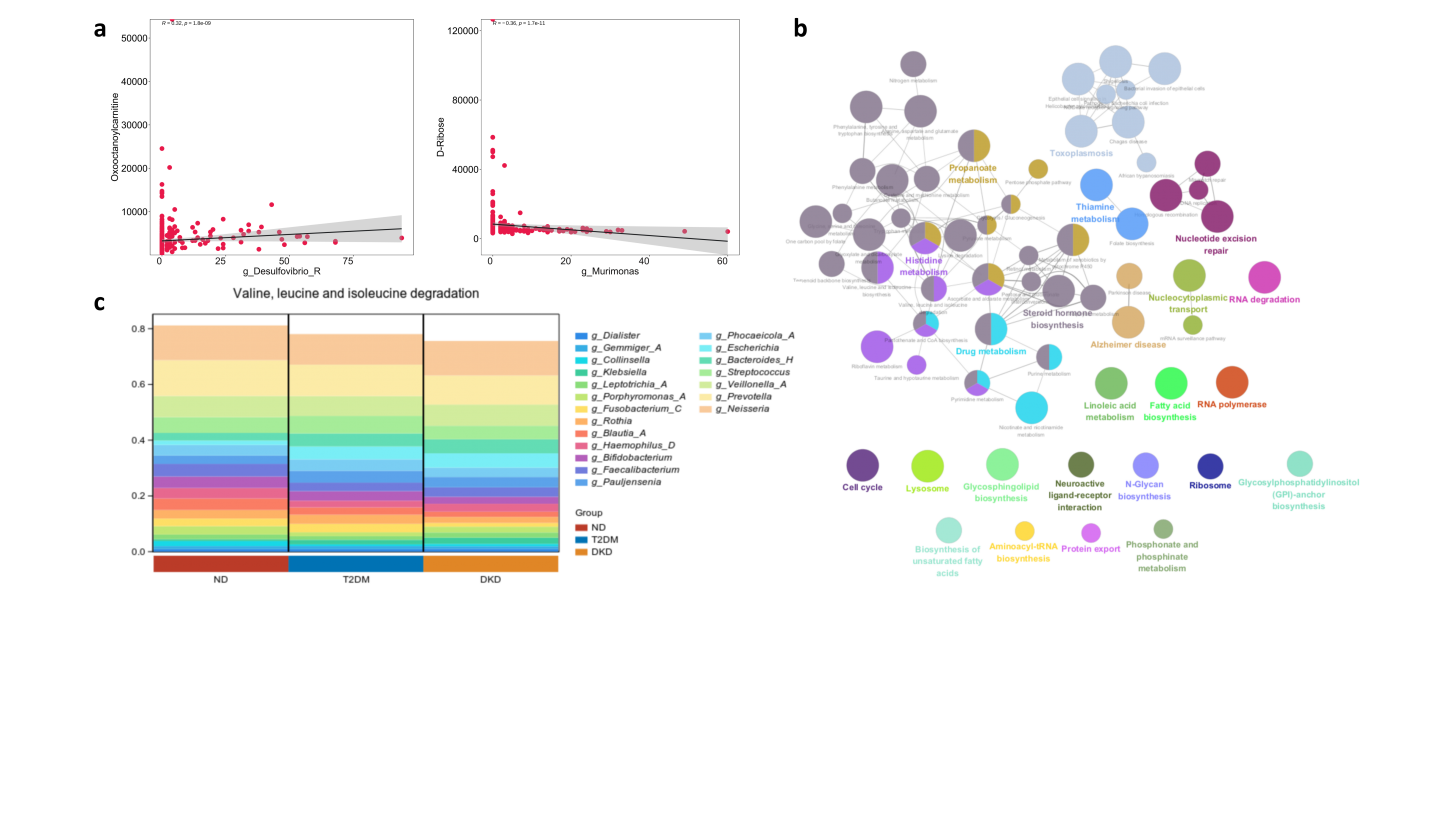
**

**Supplementary** **Fig. 5 | Multimodal analysis reveals the interaction between the microbiome and the metabolome under healthy and diseased conditions:** a. Linear correlation scatter plots showed the correlation between oral microbiota (*Desulfovibrio-R*) and metabolites(Oxooctanoylcarnitine) in the difference between T2DM and DKD(left)and the correlation between gut microbiota (*Murimonas*) and metabolites(D-Ribose) in the difference between T2DM and DKD(right) . b. The network diagram is constructed based on the correlation between metabolic pathways and microbial functions. The circles represent a specific metabolic pathway or microbial function. The color and category of the circles indicate the name of the pathway or function they represent. The size of the circles reflects the connectivity or importance of the pathway or function in the network. The lines between the circles represent the interaction relationship between the metabolic pathway and microbial function. The thickness of the lines may reflect the intensity or reliability of the interaction. c. The bar chart shows the relative abundance of the top 20 microbial genera involved in the degradation of valine, leucine, and isoleucine in different experimental groups .


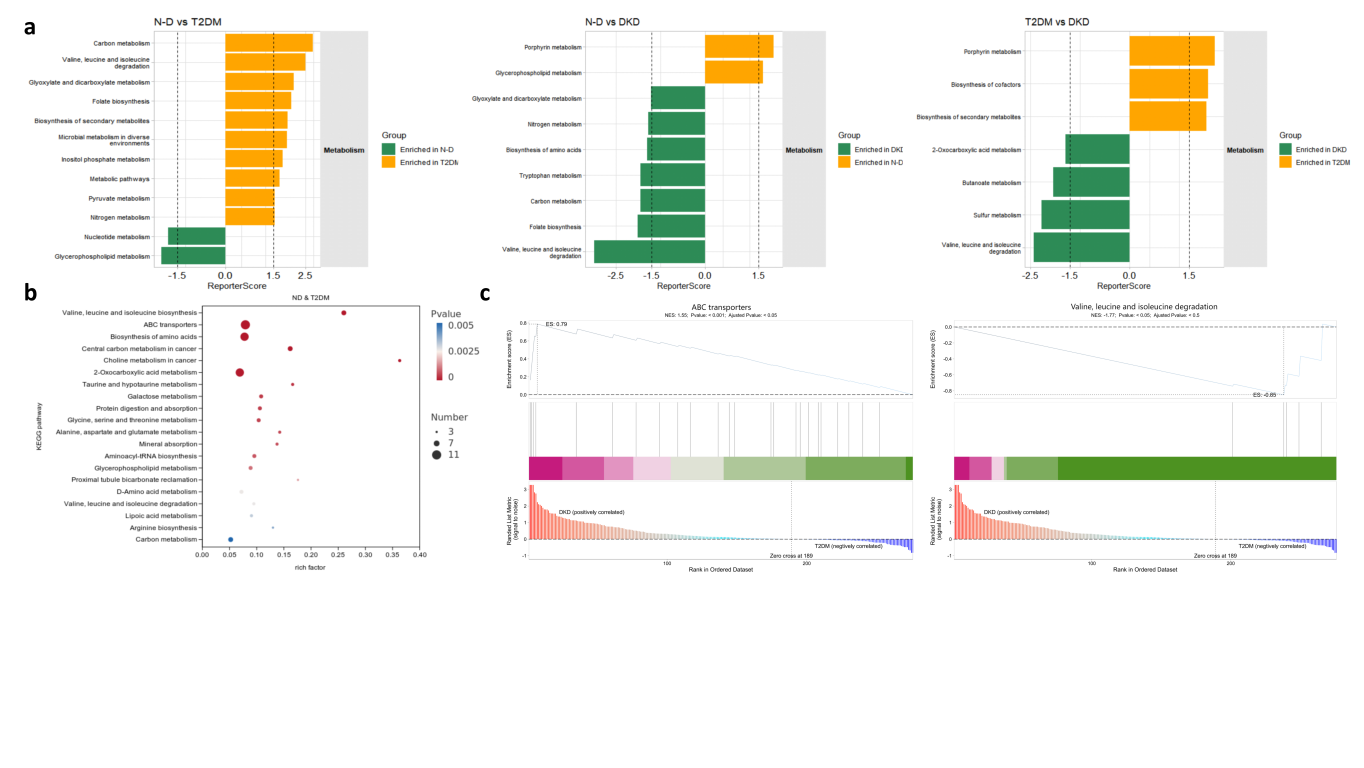


**Supplementary** **Fig. 6| Analysis of metabolic pathway differences and functions among different health states:** a. The bar charts of KEGG metabolic pathway enrichment analysis respectively show the enrichment of differentially regulated metabolic pathways among ND&T2DM, ND&DKD, and T2DM&DKD. b. The enrichment factor map shows the top 20 KEGG pathways that are most significantly enriched with differential metabolites between ND and T2DM. c. The GSEA plot shows the enrichment of ABC transporter-related gene sets (on the left) and the gene sets related to the degradation metabolism pathway of valine, leucine, and isoleucine (on the right) in the ordered dataset. The enrichment trend is displayed through the Enrichment Score (ES) curve.


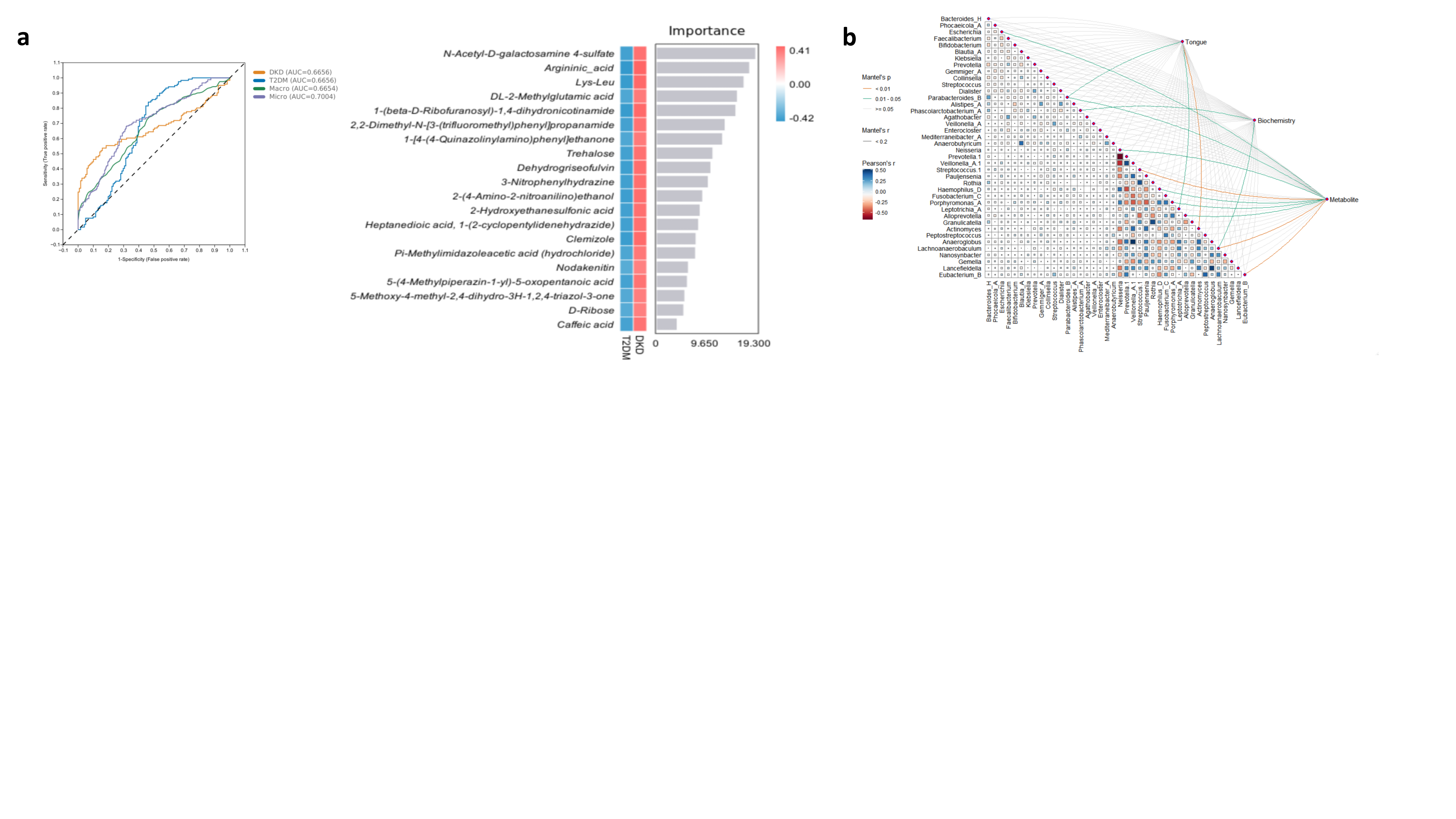


**Supplementary** **Fig. 7 | Metabolite alterations in DKD:** a. Differential marker metabolites between T2DM and DKD identified using the support vector machine (SVM) model; b. The Mantel test was employed to assess correlations between the top 20 most abundant genera across the three groups, analyzing the relationship between these factors and community composition, as well as their association with tongue objectivization, biochemical, and metabolite indices.


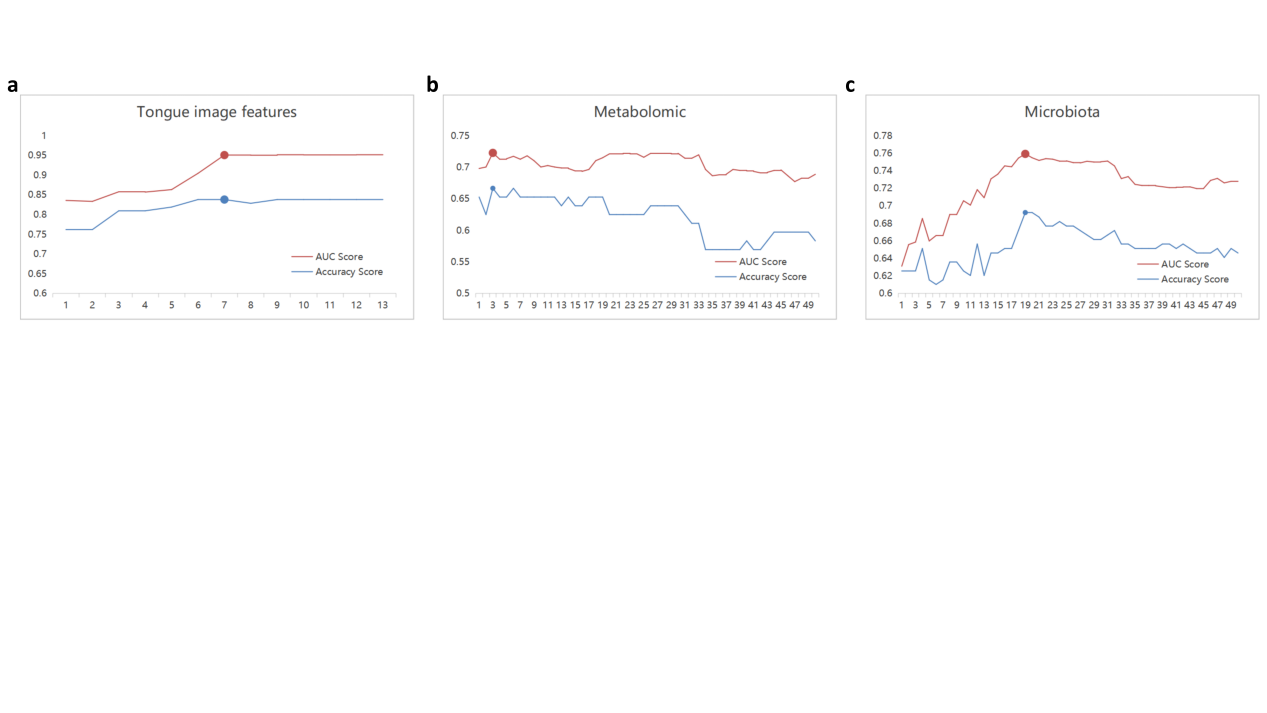


**Supplementary** **Fig. 8| The performance of different omics data in the model:** The line graph presents the performance evaluation results of the model constructed based on Tongue image features, Metabolomic and Microbiota data. Each subgraph corresponds to a different data type and shows the trend of the AUC (Area Under the Curve) score and accuracy score of the model on that data as the number of included features changes. a) Tongue image features, b) Metabolomic, c) Microbiota.
